# Focused Proteomics Analysis of Habu Snake (*Protobothrops flavoviridis*) Venom Using Antivenom-Based Affinity Chromatography Reveals Novel Myonecrosis-Enhancing Activity of Thrombin-Like Serine Proteases

**DOI:** 10.3389/fphar.2021.766406

**Published:** 2021-11-04

**Authors:** Tomohisa Ogawa, Yu Tobishima, Shizuka Kamata, Youhei Matsuda, Koji Muramoto, Masafumi Hidaka, Eugene Futai, Takeshi Kuraishi, Shinichi Yokota, Motonori Ohno, Shosaku Hattori

**Affiliations:** ^1^ Laboratory of Enzymology, Graduate School of Agricultural Science, Tohoku University, Sendai, Japan; ^2^ Graduate School of Life Sciences, Tohoku University, Sendai, Japan; ^3^ Institute of Medical Science, University of Tokyo, Kagoshima, Japan; ^4^ Department of Applied Life Science, Faculty of Bioscience and Biotechnology, Sojo University, Kumamoto, Japan

**Keywords:** antivenom, myonecrosis, phospholipase A2, proteomics, pseudoenzyme, serine protease, snake venom, venomics

## Abstract

Snakebites are one of the major causes of death and long-term disability in the developing countries due to the presence of various bioactive peptides and proteins in snake venom. In Japan, the venom of the habu snake (*Protobothrops flavoviridis*) causes severe permanent damage due to its myonecrotic toxins. Antivenom immunoglobulins are an effective therapy for snakebites, and antivenom was recently developed with effective suppressive activity against myonecrosis induced by snake venom. To compare the properties of an antivenom having anti-myonecrotic activity with those of conventional antivenom with no anti-myonecrotic activity, this study applied focused proteomics analysis of habu venom proteins using 2D gel electrophoresis. As a target protein for antivenom immunoglobulins with anti-myonecrotic activity, we identified a thrombin-like serine protease, TLSP2 (TLf2), which was an inactive proteolytic isoform due to the replacement of the active site, His43 with Arg. Additionally, we identified the unique properties and a novel synergistic function of pseudoenzyme TLf2 as a myonecrosis-enhancing factor. To our knowledge, this is the first report of a function of a catalytically inactive snake serine protease.

## Introduction

Snake envenomation is a major cause of death and long-term disability in developing countries. Approximately 5.4 million people worldwide are estimated to suffer from snakebites annually, causing around 81,000 to 138,000 deaths and around three times permanently disabilities (World Health Organization, 2021). Venomous snakes contain various bioactive peptides and proteins in their venom (Doley and Kini, 2009). Recently, we decoded the whole genome structure of the venomous habu snake (*Protobothrops flavoviridis*) and analyzed its transcriptomic data by next-generation sequencing, revealing that the production mechanisms of various venomous proteins are associated with accelerated evolution and alternative splicing (Shibata et al., 2018; Ogawa et al., 2019; Ogawa and Shibata, 2020). Although these venomous proteins, including metalloproteases (MPs), phospholipases A_2_ (PLA2s), serine proteases (SPs), C-type lectin like proteins (CTLPs), demonstrated several specific individual pharmacological activities, they also acted cooperatively and synergistically as a cocktail of toxins (Xiong and Huang, 2018). These complexes, which formed through covalent and/or non-covalent interactions of either identical (homodimers) or dissimilar (heterodimers; in some cases subunits belong to different families of proteins) subunits, exhibit much higher levels of pharmacological activity and pathophysiological effects compared to individual components during envenomation (Doley and Kini, 2009).

PLA2 (EC 3.1.1.4) catalyzes the hydrolysis of 2-acyl ester bonds of 3-*sn*-phosphoglycerides in the presence of Ca^2+^ ions to liberate 3-*sn*-lysophosphoglycerides and fatty acids. Snake venom PLA2s are major toxins and are classified into two groups (I and II) based on their disulfide bond pattern. Group I PLA2s are found in elapid snake venoms, whereas group II PLA2s are found in viperid venoms. Group II PLA2s are further divided into two subgroups: [Asp49] PLA2 and [Lys49] PLA2 forms. Several PLA2 isozymes have been identified in habu venom, including hemolytic isozumes [Asp49]PLA2 [D10070.1, D10720.1, D10722.1, AB778558.1, AB072174.1, AB778559.1], edema-inducing basic isozymes [Asp49]PLA2 (PLA-B) [D10721.1], a weak neurotoxin PLA-N [AB848131], and [Lys49]PLA2 myotoxins (BPI, BPII and BPIII) [D10718.1, D10719.1, AB470470.1] have been found (Supplementary Table S1 and Supplementary Figure S1). Moreover, SPs are major components of venom proteins. They act on various macromolecular substrates of blood coagulation, fibrinogenolysis with fibrinogen clotting activity, fibrinolytic activity including plasminogen activators, kallikrein-kinin systems, capillary permeability-increasing enzymes, and complement cascade, and on platelets, causing hemostatic defects (Seeger and Ouyang, 1979). Habu venom reportedly contains the kinin-releasing enzyme flavovilase (Komori et al., 2001), a weakly thrombin-like enzyme flavoxobin (Shieh et al., 1985) and habutobin (Sunagawa et al., 2007) that specifically release fibrinopeptide A from fibrinogen, and thrombin-like enzymes possessing fibrinopeptide A- and B-releasing activity, such as flavoviridiobin (Tatematsu et al., 2000). Flavoxobin can also act as a heterologous C3-convertase that selectively releases human C3b and C3a (Yamamoto et al., 2002). Thus, snake venom proteins possess various pharmacological activities and exert destabilizing effects on snakebite victims.

Antivenom immunoglobulins (IgGs) are one-of-a-kind effective therapies for snakebites due to the complexity and multiplicity of venom proteins (Kasturiratne et al., 2008), and were included in the WHO Essential Medicines list in 2019. In Japan, habu snakes, which inhabit the Ryukyu Islands including Okinawa and Amami/Tokunoshima Islands (Kagoshima), are dangerous pit vipers that cause severe permanent damage due to their myonecrotic toxins in their venom. Habu antivenoms are available from KM Biologics Co., Ltd. (formerly Kaketsuken Co., Kumamoto, Japan) for the treatment of habu snakebites in Okinawa and Kagoshima prefectures (Hifumi et al., 2017). However, conventional antivenoms are not always effective for myonecrosis although they are proven effective treatment for lethality and hemorrhagic lesion caused by habu snake toxin. More recently, antivenom (anti-serum) was developed that can effectively suppress myonecrosis caused by habu crude venom (Table 1). The aim of the current study was to elucidate the properties of an anti-serum with anti-myonecrotic activity compared with those of a conventional antivenom without anti-myonecrotic activity, by conducting focused proteomics analysis of habu venom proteins. The study findings will help develop new antivenom treatments.

**TABLE 1:** Anti-pathological suppressing effect of antivenoms.

Venom dose (μg)	Antivenom		Autopsy view			Pathology organization views		
	No.	dose	Hemorrhage	edema	swelling	Hemorrhage	myonecrosis	cellular infiltration
20		-	+++	+++	+++	+++	+++	++
20	34	10 μL	-	-	-	-	++	+++
20	1,469	10 μL	-	+	-	-	-	+

## Materials and Methods

### Materials

Habu (*Protobothrops flavoviridis*) crude venom was provided by the Kagoshima Prefectural Public Health Office in Japan. *P. flavoviridis* [Asp49] PLA2 and BPII were prepared from crude venom as previously reported with some modifications (Tanaka et al., 1986; Liu et al., 1990). Sephadex G-75, a Hi-Trap NHS-activated HP column (1 ml) and a HiTrap benzamidine column (1 ml) were obtained from GE Healthcare Ltd. (Buckinghamshire, United Kingdom). CM-52 was purchased from Whatman (Maidstone, Kent, United Kingdom). POROS HS was obtained from Life Technologies (Carlsbad, CA, United States). Phenylmethylsulfonyl fluoride (PMSF) and RPMI-1640 were purchased from Sigma-Aldrich Co. (St. Louis, MO, United States). Dishes and 96-well microtiter plates were purchased from Becton Dickinson Co. (Franklin Lakes, NJ, United States). Centrifugal filter devices and ZipTip C18 pipette tips were obtained from Merck Millipore (Darmstadt, Germany). The Silver Stain MS Kit and V8 protease were purchased from Wako Pure Chemical Industries, Ltd. (Osaka, Japan). The XL-trypKit was purchased from APRO (Osaka, Japan). Boc-Val-Pro-Arg-MCA was purchased from Peptide Laboratory (Osaka, Japan). SkMCs (human skeletal muscle origin cells) and their basal medium (SkBM) were purchased from Lonza Ltd. (Visp, Switzerland). SW839 (human kidney origin) cells were obtained from the Cell Resource Center for Biomedical Research (Institute of Development, Aging and Cancer, Tohoku University, Sendai, Japan). The Cell Counting Kit-8 was purchased from DOJINDO Lab, Inc., (Kumamoto, Japan). 3,3′,5,5′-tetramethylbenzidine was purchased from Tokyo Chemical Industry Co. Ltd. (Tokyo, Japan). All other reagents were of commercial grade and were purchased from Wako Pure Chemicals or Nacalai Tesque (Kyoto, Japan).

### Antivenoms

Two types of antivenom against habu venom, one without anti-myonecrotic activity (lot no.34 (manufacture’s serial number)) and one with anti-myonecrotic activity (lot no.1469), were obtained from the Kagoshima Prefecture government’s pharmaceutical affaires section and KM Biologics Co., Ltd. (formerly Kaketsuken or The Chemo-Sero-Therapeutic Research Institute, Kumamoto, Japan), respectively. Conventional antivenom (lot no.34) without anti-myonecrotic activity, which is a mixture of some horse sera immunized by different venoms derived from Okinawa habu or Amami one, is a product approved by the Japanese Pharmacopoeia (approved number: 61E1174, expiry dates: January 20, 2013, >300 units/ml for anti-hemorrhage activity (anti-HA)) as pharmaceutical agent for habu snake bite, Freeze-dried Habu Antivenom “Kaketsuken.” On the other hand, antivenom with anti-myonecrotic activity (lot no.1469) was newly prepared from serum of a female horse immunized by antigen injection as follows; preliminary twice immunization by toxoid (formalin-inactivated crude venom) with incomplete Freund’s adjuvant, subsequently, after for 7 weeks breaking, eight times booster immunization by increasing the dose of Amami habu crude venom (3–320 mg). To assess antibody titers of antivenoms against myonecrotic [Lys49] PLA2, ELISA test was conducted by using BPII ([Lys49] PLA2) and [Asp49] PLA2 as antigens. Briefly, purified antigens BPII and [Asp49] PLA2 (625 ng/well each) were respectively immobilized on the wells of a microtiter plate for 2 h at room temperature (RT), and blocked with 1% bovine serum albumin (BSA). After washing with phosphate buffered saline (PBS: 10 mM Na_2_HPO_4_, 1.8 mM KH_2_PO_4_, 2.7 mM KCl, 137 mM NaCl, pH7.4), 100 μL of serial diluted antivenoms (500–64,000 dilutions) were added on the microtiter plate wells, and were incubated at 4°C overnight. After washing with PBS three times, 100 μL of secondary antibody, horseradish peroxidase (HRP)-labeled goat anti-equine IgG (H + L) (Proteintech Japan, Tokyo), was added on plate wells, and incubated for 2 h at RT. After washing the plate four times with PBS, 100 μL of 3,3′,5,5′-tetramethylbenzidine substrate in 0.2 M citrate buffer (pH 4.0) including 0.01% H_2_O_2_ (substrate for HRP) was dispensed on the plate wells, and then monitored sufficient color development absorbance at 450 nm after adding 100 μL of the stop solution (1 M H_2_SO_4_).

### Animal and Animal Experiments

DDY mice (6 weeks-old) were purchased from CLEA Japan, Inc. (Tokyo, Japan). The mice were maintained for 1 or 2 weeks and had free access to the laboratory animal standard diet and tap water. *Protobothrops flavoviridis* crude venom (CV, 20 μg) with/without 10 μL of antivenoms (lot no. 34 or lot no. 1468) (total volume: 15 μL) were injected into musculus gastrocnemius of mice left hindlimb, and the control group was injected with only saline. After 24 h, the mice were euthanized, and the hindlimbs were dissected for tissue preparation. *In vivo* experiments using BPII and TLf2 were also conducted same as crude venom and antivenoms described above. Thereafter, the tissue samples were dehydrated in a graded ethanol series and embedded in paraffin. 5 μm sections were cut in a microtome and stained with hematoxylin and eosin to be examined under the light microscope. The experimental plans used in this work were reviewed and approved by the Animal Resarch-Animal Care Committee of the Institute of Medical Science, the University of Tokyo or Graduate School of Agricultural Sciences, Tohoku University (approved number: 21BA-7). These procedures are in accordance with standards outlined by Japanese laws for use of experimental animals (Act on Welfare and Management of Animals), and with ethical principles adopted by the University of Tokyo and Tohoku University.

### Focused Proteomics of Habu Venom Proteins Using Antivenom-Conjugated Affinity Column

The IgGs from the two habu antivenoms, which were obtained by equine immunization with habu venom, were individually purified using protein G columns and then coupled overnight at 4°C using HiTrap NHS-activated HP columns with coupling buffer (0.2 M NaHCO_3_ containing 0.5 M NaCl, pH 8.3). After washing with 4 ml blocking buffer (0.5 M ethanolamine containing 0.5 M NaCl, pH 8.3), followed by 4 ml washing buffer (0.1 M sodium acetate containing 0.5 M NaCl, pH 4.0), the columns were equilibrated with 5 ml PBS. The binding capacity of each column (2.0 mg/ml) was estimated by monitoring the adsorbed peak area measurement for serial concentrations of crude venom proteins.

Crude venom (2 mg) was dissolved in 2 ml PBS (pH 7.4) and applied to both habu antivenom IgG (lot no. 34)- and antivenom IgG (lot no. 1469)-conjugated columns. The columns were washed with PBS (pH 7.4) buffer and eluted with 0.1 M glycine-HCl (pH 2.7) buffer at a flow rate of 1 ml/min. The eluted protein fractions were neutralized by adding the 1/10 volume of 1 M Tris-HCl (pH 8.5). Flow-through and adsorbed peak fractions were individually dialyzed against distilled water and lyophilized.

For proteomics analysis using two-dimensional polyacrylamide gel electrophoresis (2D-PAGE), the proteins separated by the antivenom IgG-conjugated affinity columns were analyzed by isoelectric focusing (IEF) using the IPGphor System (Amersham Biosciences, Piscataway, NJ, United States) with immobilized pH gradient (IPG) strips (pI 3–11), and then subjected to the sodium dodecyl sulfate polyacrylamide gel electrophoresis (SDS-PAGE) under reducing conditions. SDS-PAGE was performed on 15% slab gels according to the method described by Laemmli. The Auto 2D BM-100 device (SHARP CO., Ltd., Osaka, Japan) was also used to conduct 2D-PAGE analysis using pI 3–10 and 12.5% gels. A typical condition for IEF on Auto 2D BM-100 device was 5 steps (Step 1: 200 V for 5 min, Step 2: 200–1000 V linear gradient for 5 min, Step 3: 1000 V for 5 min, Step 4: 1,000–6000 V linear gradient for 10 min, Step 5: 6000 V for 5 min) at 100 μA, at 10°C. After running 2D-PAGE, the protein spots were stained with Oriole fluorescent gel staining (Bio-Rad Lab. Inc.) or using the Silver Stain MS Kit. Proteins were quantified and compared using CS analyzer ver. 3.0 and ImageSaver5 for EZ-Capture MG (ATTO Co., Tokyo, Japan).

The Silver-stained spots on the gel were cut into 1 mm^3^ pieces, washed with 100 μL of De-staining solution (in Silver Staining MS Kit, Wako Pure Chemicals), incubated with same solution at RT for 15 min with gentle agitation, washed twice with 500 μL of 0.1% trifluoroacetic acid (TFA) (15 min each), and then dehydrated by incubating the gel pieces with 100 μL acetonitrile at RT for 5 min. After removing the acetonitrile, the gel pieces were dried for 15 min *in vacuo*, and then reduced with 10 mM DTT in 25 mM ammonium bicarbonate buffer for 2 h at 56°C. After cooling to RT, the gel pieces were washed with 100 μL of 25 mM ammonium bicarbonate buffer and treated with 100 μL of 55 mM iodoacetamide. The gel pieces were then washed with 100 μL of 0.1% TFA for 10 min and twice dehydrated by treatment with 50% acetonitrile in 25 mM ammonium bicarbonate buffer. The gels were dried for 15 min *in vacuo*, and subsequently digested overnight at 37°C with trypsin using the XL-trypKit or V8 protease in 0.1 M NH_4_HCO_3_ containing 4 M urea. Then, 50 μL of extraction buffer solution (50% acetonitrile containing 5% TFA) was added to the gel pieces, which were incubated for 30 min at RT with shaking to extract the digests. After concentrating the digests, the samples were desalted using ZipTip C18 pipette tips according to the manufacturer’s instructions. The desalted samples (1 μL) were mixed with α-cyano-4-hydroxycinnamic acid matrix and then applied to a MALDI-TOF MS plate. Samples were analyzed by MALDI-TOF MS/MS reflector positive mode using the TOF/TOF™ 5800 Analyzer (ABSCIEX, Framingham, MA, United States) with mass range from 800.00 to 4,000.00 Da. *In gel* digested samples were also analyzed by nanoLC-MALDI TOF MS/MS using a DiNa Nano LC system equipped with a DiNa MALDI spotting device (KYA Technologies Co., Tokyo, Japan). Molecular masses were calibrated using the Sequazyme Peptide Mass Standards Kit (Applied Biosystems). Protein identification was performed by searching of each MS/MS spectrum against the protein sequence databases derived from the RNA-seq data of *P. flavoviridis* snake venom by using Protein Pilot software (version 3.0; AB Sciex) with the Paragon method.

Publicly available datasets were analyzed in this study. Protein profiling data can be found in the JPOST repository, a member of ProteomeXchange, with ID numbers of JPST001309/PXD028215 and JPST001307.

### Purification of Thrombin-like Serine Proteases, TLf1 and TLf2, From Habu Venom

To isolate the two thrombin-like serine proteases, TLf1 and TLf2, the habu snake crude venom was separated by cation exchange chromatography, as previously reported with some modifications (Shieh et al., 1985). Briefly, habu crude venom was loaded onto CM-52 column (ϕ 15 mm × 870 mm) equilibrated with 20 mM ammonium acetate (pH 6.8) and eluted with a linear gradient to 500 mM ammonium acetate (pH 6.8) (Supplementary Figure S2). Flavoxobin (TLf1) obtained in the acidic flow-through peak fraction of the CM-52 column was further purified by affinity chromatography on the Hi-trap benzamidine Sepharose FF column (Supplementary Figure S3). The most basic peak fraction, which included TLf2, was further purified by cation-exchange chromatography on a POROS HS column (7.5 × 200 mm), and eluted with a gradient of 0–1 M NaCl in 50 mM Tris-HCl (pH 8.8) (Supplementary Figure S4). The N-terminal amino acid sequence of purified TLf2 was confirmed by gas-phase protein sequencing using the Shimadu PPSQ-10 Protein Sequencer (Shimadzu, Kyoto, Japan). Purified TLf1 was confirmed by MALDI-TOF MS/MS analysis with trypsin digestion (Supplementary Figure S5).

### Assay for Serine Protease Hydrolytic Activity of TLf2

The hydrolytic activity of TLf2 was determined by incubation with fibrinogen (8.6 mg/ml) in 20 mM Tris-HCl buffer (pH 7.5) containing 0.15 M NaCl for 30 min and 24 h at 25°C. To assess fibrinogen degradation, SDS-PAGE was performed using a 15% separating gel and the protein bands were stained with Coomassie brilliant blue R-250. Furthermore, the hydrolytic activity of TLf2 was also analyzed using a synthetic fluorogenic substrate, Boc-Val-Pro-Arg-MCA (200 μM), in a 96-well black microtiter plate. Fluorescence changes, based on the amount of amino methyl coumarin (AMC) released, were monitored using a Gemini XPS-TON fluorescence microplate reader (Molecular Devices Japan, Tokyo, Japan). The excitation and emission wavelengths were 365 and 440 nm, respectively. Human α-thrombin (Mochida Pharmaceutical Co. Ltd., Tokyo, Japan) was used as a positive control.

### Cell Culture and Assay for Cytotoxicity of TLf1, TLf2, and BPII

SW839 and SkMC cells were cultured in 90 mm dishes containing 10 ml of RPMI-1640 supplemented with 10% fetal bovine serum (for SW839) and SkBM medium (for SkMC) at 37°C under an atmosphere of 5% CO_2_. After reaching 80% confluence, the cells were detached from the dish by trypsin-EDTA treatment and plated on a 96-well microtiter plate at approximately 1.0 × 10^5^ cells/well. These cells were cultured under the same conditions described above for 24 h to approximately 70% of confluence prior to the cytotoxicity assay.

The proteins were dissolved in PBS buffer, and subsequently passed through a sterile 0.22 μm filter. The effects of TLf2 and TLf1 on the cytotoxic activity of [Lys49] PLA2 (BPII) were analyzed by incubating SW839 and SkMC cells with various concentrations of TLf2 and TLf1 mixed with 2.5 μg/ml and 25 μg/ml BPII. After incubation for 24 h, cell viability was analyzed using the Cell Counting Kit-8, measuring the absorbance at 450 nm using an iMark microtiter plate reader (Bio-Rad Laboratories, Hercules, CA, United States). All measurements were performed in triplicate. The data were evaluated by using Student’s t-test.

### Protein-Protein Binding Analysis by Surface Plasmon Resonance

The BIAcore™ surface plasmon resonance (SPR) instrument (Amersham Biosciences) was used to determine the interaction between the TLfs and BPII. Purified TLf2 and TLf1 were diluted to a concentration of 100 μg/ml in 10 mM HEPES buffer (pH 7.5) and immobilized on the carboxymethylated dextran-modified gold surface of a CM5 sensor chip via primary amino groups using carbodiimide chemistry as described in the manufacturer’s protocol. Briefly, the carboxyl groups on CM5 were activated by 10 mM *N*-hydroxy succinimide and 400 mM *N*-ethyl-*N*’-(3-diethylaminopropyl)-carbodiimide, followed by the addition of 20 μL of TLf2 or TLf1. The remaining activated groups were blocked by addition of ethanolamine. BPII and [Asp49] PLA2 were individually dissolved in HBS (10 mM HEPES buffer (pH 7.4) containing 0.15 M NaCl, 3 mM EDTA, and 0.005% (v/v) surfactant P-20) at a concentration of 200 μg/ml. Then, 2–16 serial solutions were injected onto the TLf1 or TLf2-immobilized surfaces and unmodified sensor chip CM5 (control) at a flow rate of 20 μL/min. The sensor chip surface was regenerated with 10 mM HCl (40 μL) at the end of each measurement. Sensor-grams were analyzed using nonlinear least-squares curve fitting with BIAevaluation software (Amersham Biosciences).

### In Silico Analysis of the Interactions Between TLfs and BPII

Interactions between TLfs (TLf1 and TLf2) and BPII were estimated by the protein-protein docking simulation using the HDOCK server that based on hybrid docking strategies, template-based modeling, and *ab initio* docking (Yan et al., 2020). The 3D structures of TLf1 and TLf2 were predicted by homology modeling based on the complex 3D structure of trypsin and its inhibitor (PDB 1TX6). Predicted 3D structures were used as receptor molecules, while [Lys49] PLA2 (BPII, PDB 6AL3) (Matsui et al., 2019) was used as a ligand molecule. The top 10 predicted models with the lower docking energy scores were obtained for each interaction.

### Statistical Analysis

Statistical analysis was carried out using Student’s *t*-test at a 99% confidence level.

## Results

### Identification of Target Proteins Adsorbed to Anti-Myonecrosis-type Antiserum-Conjugated Column

**FIGURE 1:**
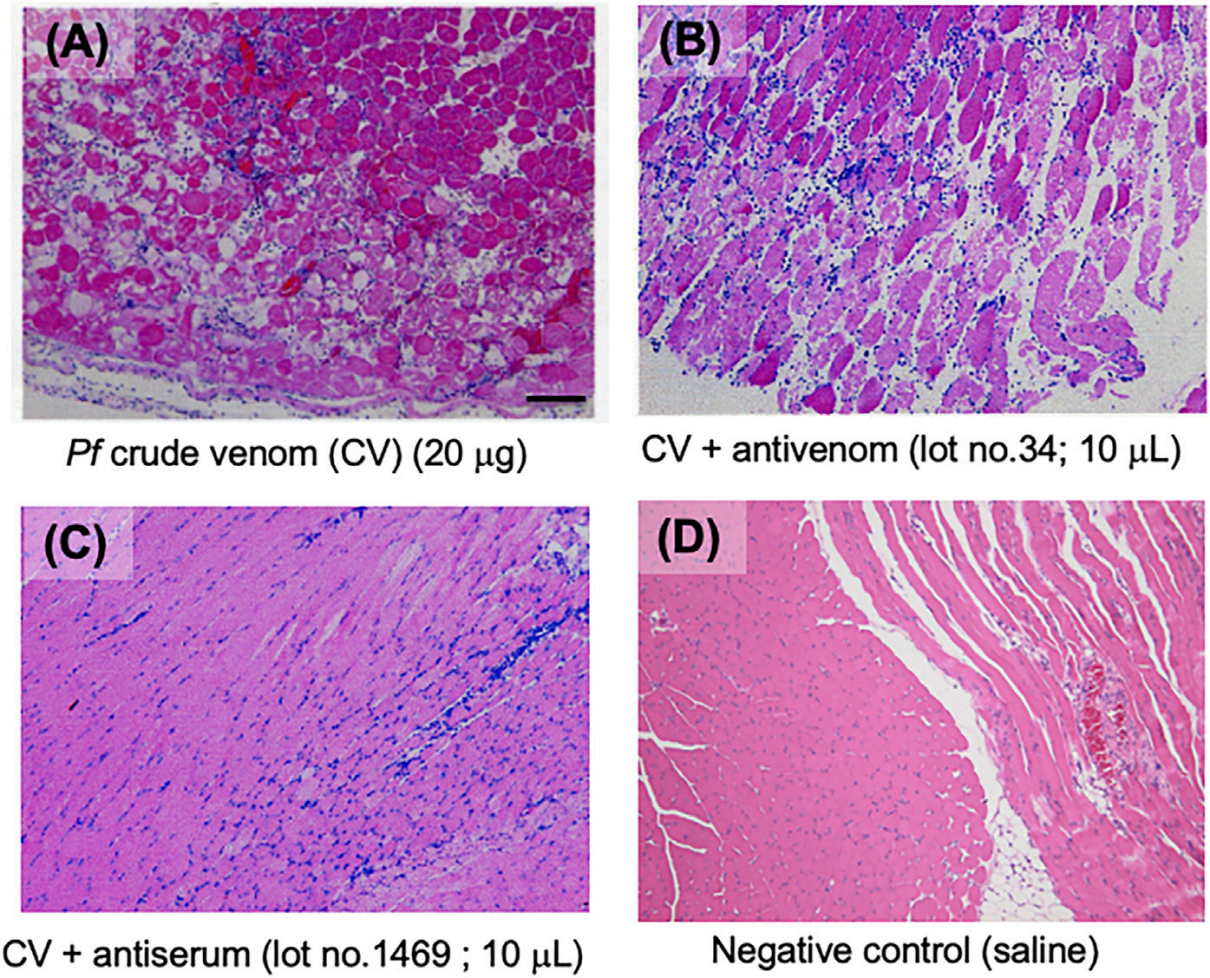
Anti-necrosis activities of the antivenom. Haematoxylin and eosin (H&E)-stained sections of mice femoral region injected with habu crude venom **(A)**, crude venom + antivenom (lot no. 34) **(B)**, and crude venom + antivenom (lot no. 1469) **(C)** were analyzed histologically under a light microscope. All images are the same magnification. Scale bar = 100 μm.

**FIGURE 2:**
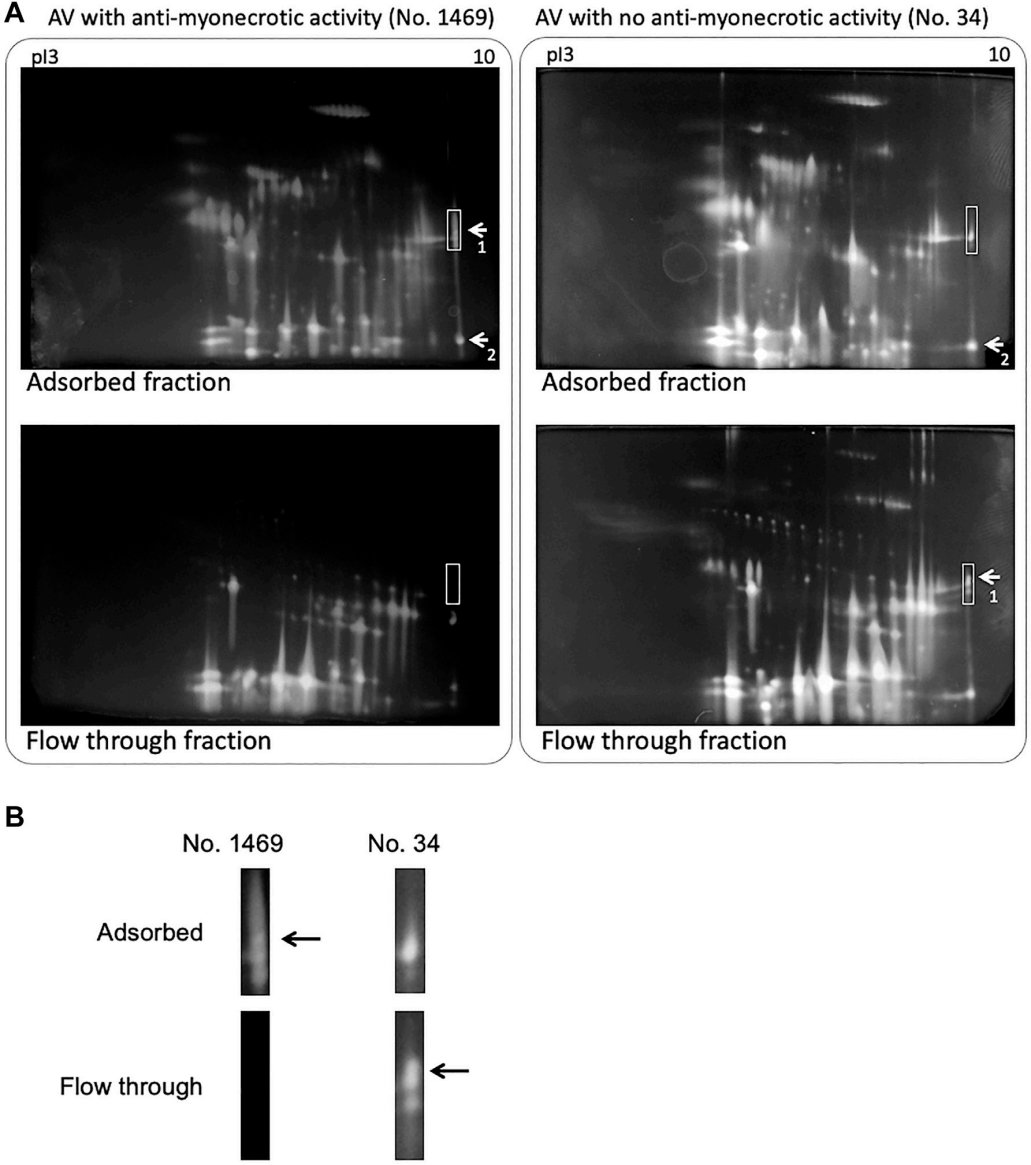
2D PAGE of habu crude venom separated by antivenom (AV)-conjugated affinity chromatography. **(A)** 2D PAGE profiles of adsorbed **(upper)** and flow through fractions **(down)** on antivenom-conjugated column using lot no. 1469 **(left)** and lot no. 34 **(right)** antivenoms, respectively. Samples were loaded on the Auto 2D BM-100 device using pI 3–10 and 12.5% gels. The protein spots were stained using the Silver Stain MS Kit. The labeled spots 1 and 2 indicated TLf2 and BPII proteins, respectively. Basic pI regions boxed with white lines were enlarged **(B)**. Arrows indicated TLf2 spot.

The pathological diagnosis and autopsy in mice including anti-myonecrosis activities of two types of habu antivenoms were determined against habu crude venom (Table 1). Newly prepared anti-venom (lot no. 1469) showed anti-lethal activity (anti-LA) and anti-HA of immunized serum with 676 units/ml (for anti-LA), 550 units/ml (for anti-HAI (HR1a/HR1b)), and 233 units/ml (for anti-HAII (HR2a/HR2b)), respectively (unpublished data). Although both anti-venoms had anti-hemorrhage activities (>300 units/ml), they showed very different effect on myonecrosis caused by [Lys49]PLA2 myotoxins. Figure 1 presents histochemical evidence supporting the effectiveness of the anti-myonecrotic antivenom in mouse skeletal muscle treated with habu venom, in which lot no. 1469 (with anti-myonecrotic activity) prevented the necrosis of mouse fascia lata tissues, while lot no. 34 (without anti-myonecrotic activity) exerted no protective effects. First, the antibody titration of two different antivenoms, lot no. 1469 with anti-myonecrotic activity and lot no. 34 without anti-myonecrotic activity, were assessed by using ELISA against BPII, one of the [Lys49]PLA2 myotoxins in habu venom, indicated that the antivenom (lot no. 1469) with anti-myonecrotic activity showed the higher (about ten times) sensitivity against BPII compared with conventional antivenom, lot no. 34, although both antivenoms showed the similar reactivity against [Asp49]PLA2 (Supplementary Figure S6). Next, to elucidate the different properties of the two antivenoms, including their target venom proteins, comparative proteomics analysis of habu venom proteins was conducted using antivenom IgG-conjugated affinity columns. 2D-PAGE analysis demonstrated similar profiles for both venom proteins purified by antivenom-columns in both adsorbed and flow through fractions (Figure 2). Both antivenom-specific-conjugated columns bound necrotic [Lys49] PLA2 at similar level (indicated by arrows labelled 2 in Figure 2A), of which partial amino acid sequences were determined by MALDI-TOF MS/MS analysis as shown in Supplementary Figure S7. However, some distinct differences were detected in the adsorbed fraction of the antivenom with anti-necrotic activity (lot no. 1469), while disappeared in the flow-through fraction, including a ∼30 kDa protein with a basic pI ranging from 9 to 10 (Figure 2). Interestingly, this protein was detected in the flow-through fraction of the antivenom without anti-myonecrotic activity (lot no. 34) (Figure 2B), and confirmed by reproducibility experiment using different 2D-PAGE condition (pI 3–11, 15% gel) (Supplementary Figure S8). These results indicated that the basic ∼30 kDa protein showed specific binding to the antivenom that prevented necrosis (lot no. 1469).

To investigate the venom proteins in more detail, we separated the basic 30 kDa protein by combined cation-exchange chromatography on a CM-52 column (Supplementary Figure S2) and further purified the protein by affinity chromatography (Supplementary Figure S9A). The SDS-PAGE profiles of the fractions from the two antivenom-specific-conjugated columns (Supplementary Figure S9B) indicated the 30 kDa protein more effectively bound to anti-myonecrotic antiserum than that without anti-myonecrotic activity.

### Amino Acid Sequence and Molecular Properties of the 30 kDa Protein That Specifically Bound to Anti-myonecrotic Antivenom

**FIGURE 3:**
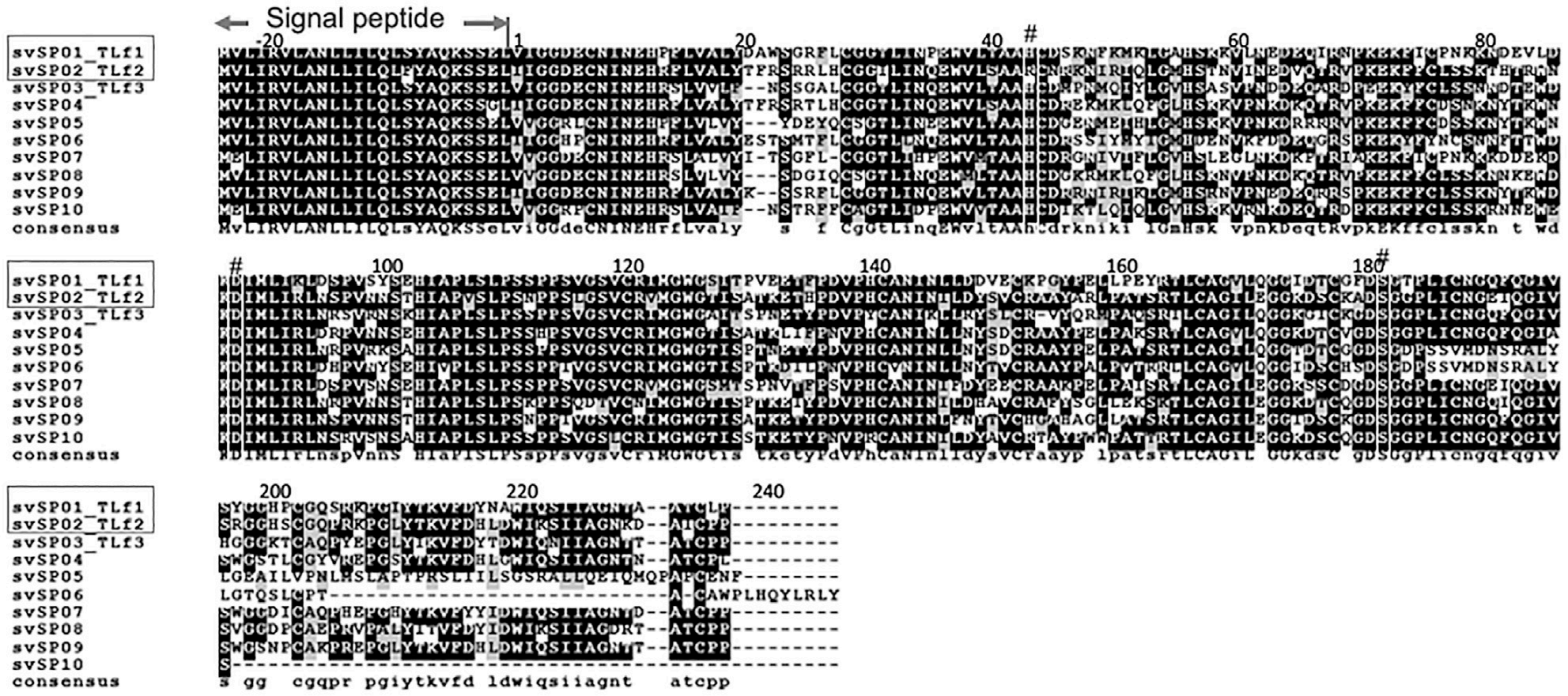
Amino acid sequence alignment of *Protobothrops flavoviridis* serine proteases identified from genome sequence. Multiple sequence alignment was conducted by CLUSTALW (ver. 2.1) on DDBJ web site at http://clustalw.ddbj.nig.ac.jp/using amino acid sequences of *P. flavoviridis* serine proteases, svSP01∼svSP10, identified from genome data (Shibata et al., 2018) and represented by using BOXSHADE 3.21. svSP01_TLf1 is Flavoxobin, and svSP02_TLf2 is inactive serine protease isoform, of which active site His43 was replaced by Arg. The symbol # indicates the catalytic triad, Asp88, His43, and Ser182.

The N-terminal sequence analysis of the purified basic 30 kDa protein was determined to be IIGGDEXNINEHRFLVALYXF, and MALDI TOF-MS/MS analysis with V8 protease and trypsin digestion, respectively, covered 47.9% of the full-length amino acid sequence of the 30 kDa protein (Supplementary Table S2 and Supplementary Figure S10). These results indicated the purified 30 kDa protein was identical to the basic serine protease homolog, TLf2, of which the amino acid sequence was previously determined from cDNA cloning (Deshimaru et al., 1996). The amino acid sequence of TLf2 suggested it to be an inactive form of serine protease with a mutation at one of the catalytic triad residues, His43, by arginine (Figure 3).

**FIGURE 4:**
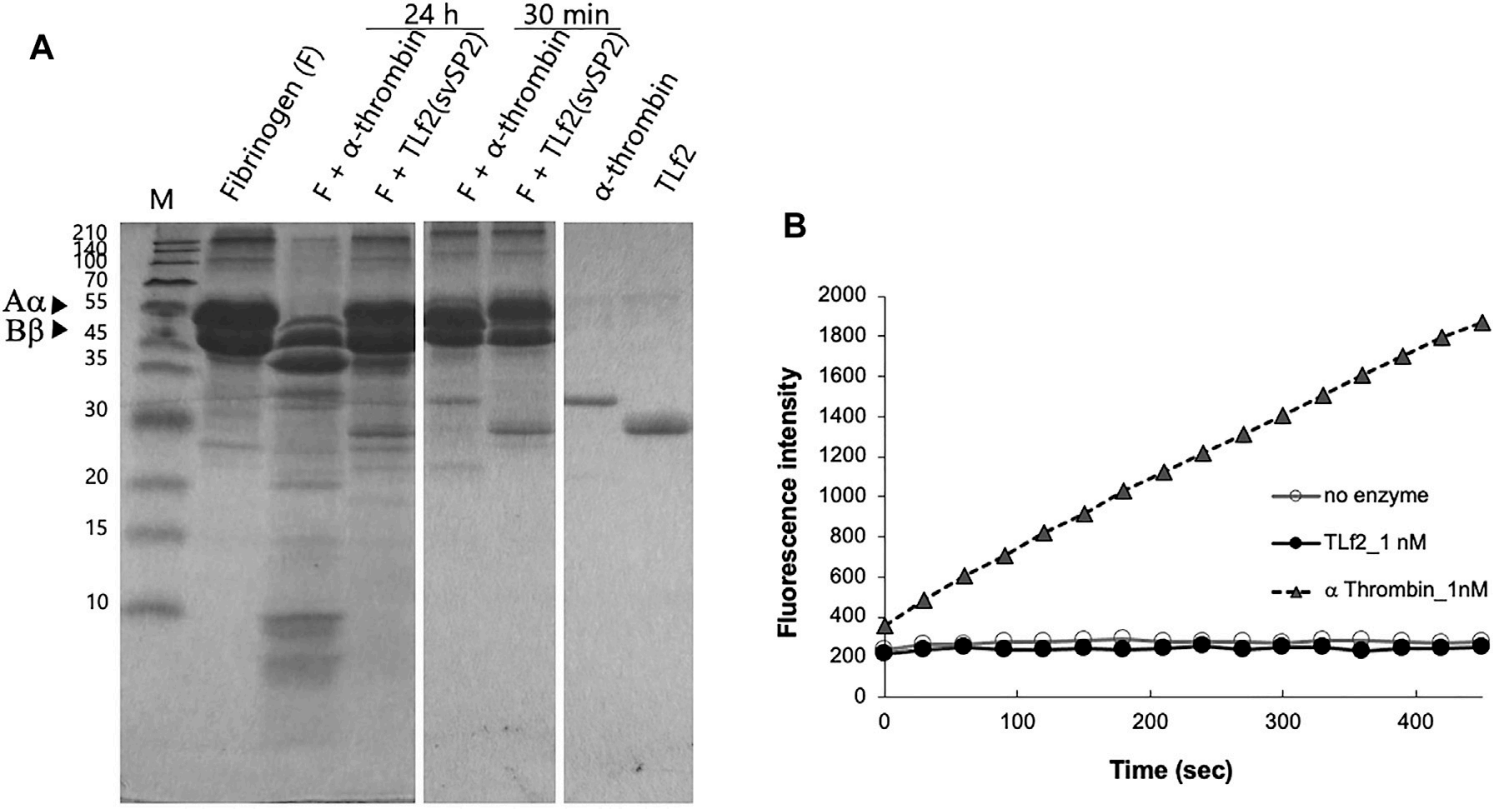
Coparison of enzymatic activity of TLf2 with α thrombin on the fibrinogen **(A)** and synthetic substrate **(B)**. **(A)** Fibrinogen (F) was loaded on SDS-PAGE after incubating with/without TLf2 or α thrombin for 30 min or 12 h. **(B)** Synthetic fluorogenic substrate, Boc-Val-Pro-Arg-MCA (200 μM), was used. Negative control was carried out without enzyme.

To confirm whether TLf2 was inactive, the hydrolytic activity of TLf2 was assayed using fibrinogen and a synthetic fluorogenic substrate, and compared with that of human α-thrombin. The SDS-PAGE profiles of fibrinogen treated with/without TLf2 or human α-thrombin indicated that TLf2 displayed no catalytic activity against fibrinogen after incubation for 24 h (Figure 4A). Additionally, TLf2 displayed no enzymatic activity against the synthetic fluorogenic substrate (Figure 4B). Thus, replacement of the catalytic triad amino acid, His43 in TLf2 with Arg rendered the serine protease inactive.

### Effects of TLf2 and Flavoxobin (TLf1) on Necrosis Activity of [Lys49] PLA2

**FIGURE 5:**
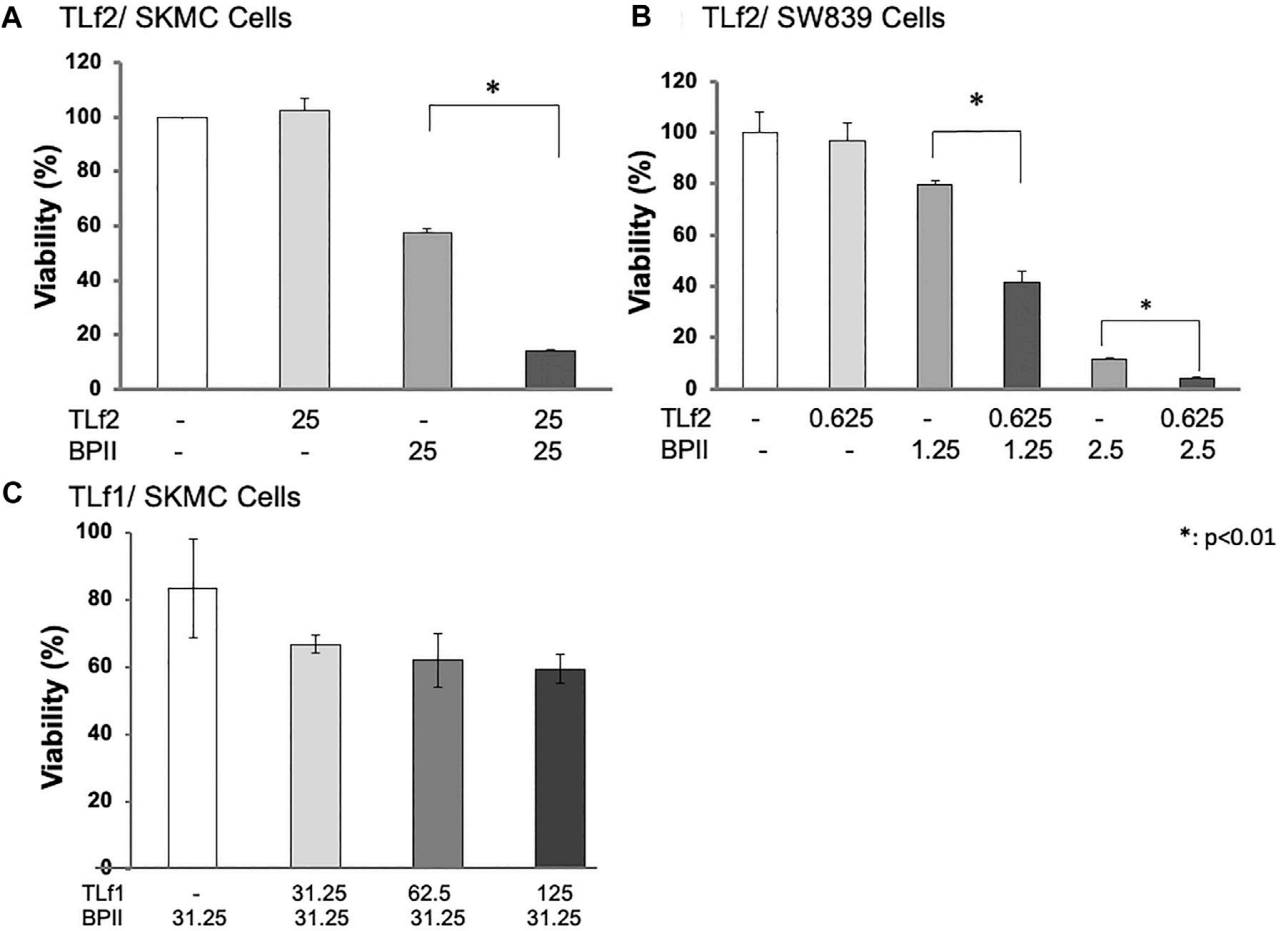
Synergic enhancing activity of TfL2 and TLf1 on cytotoxicity of BPII against SkMC **(A, C)** and SW839 cells **(B)**. SW839 and SkMC cells were inoculated into 96-well microtiter plates (1.0 × 10^5^ cells/well), and treated with [Lys49] PLA2 (BPII) and TLf2 or TLf1 for 24 h at various concentrations (μg/mL). Cell viabilities were analyzed using Cell Counting Kit-8 (WST-8). All measurements were conducted in triplicate. The data were evaluated by using Student’s t-test. *: *p* < 0.01.

TLf2 was shown to bind specifically to anti-myonecrotic antivenom, which efficiently suppressed the myonecrosis of skeletal muscle caused by habu crude venom (Figure 1). This result suggested that TLf2 possessed necrotic activity by itself and/or affected necrosis factors, such as [Lys49] PLA2 (BPII). Therefore, we aimed to confirm the cytotoxicity of TLf2 on SW839 and SkMC cells; however, TLf2 exerted no toxicity on either cell line (Figure 5). Next, we analyzed the effect of TLf2 on necrosis factor [Lys49] PLA2 (BPII). Interestingly, TLf2 drastically decreased the survival rate of BPII-treated cells compared to those treated with BPII alone (Figures 5A,B). The number of viable cells treated with both TLf2 and BPII was significantly decreased by ∼50 and 25% in SW839 and SkMC cells (*p*-values <0.01), respectively, compared with cells treated with BPII alone (Figures 5A,B). These results indicated that TLf2 exerted a synergistic enhancing effect on myonecrotic [Lys49] PLA2, representing a novel function. TLf1 (flavoxovin), a major thrombin-like serine protease in habu snake venom, was further assessed for its ability to enhance [Lys49] PLA2 myotoxicity and elucidate whether the myonecrosis-enhancing activity was unique to TLf2 or common to other thrombin-like serine proteases. As shown in Figure 5C, TLf1 alone had no effect on cells, but it exerted a weak synergistic effect on myonecrosis caused by BPII compared with TLf2. These results indicated that the synergistic pharmacological activity of TLf2 was unique.

Further, synergistic cytotoxic effects of TLf2/TLf1 against BPII were provided a preliminary *in vivo* assessment using mice. Although both TLf2 and TLf1 showed the synergistic effects compared to the treating with BPII alone, noticeable difference between TLf2 and TLf1 could not be observed (Supplementary Figure S11).

### Interactions Between TLfs and PLA2s

**FIGURE 6:**
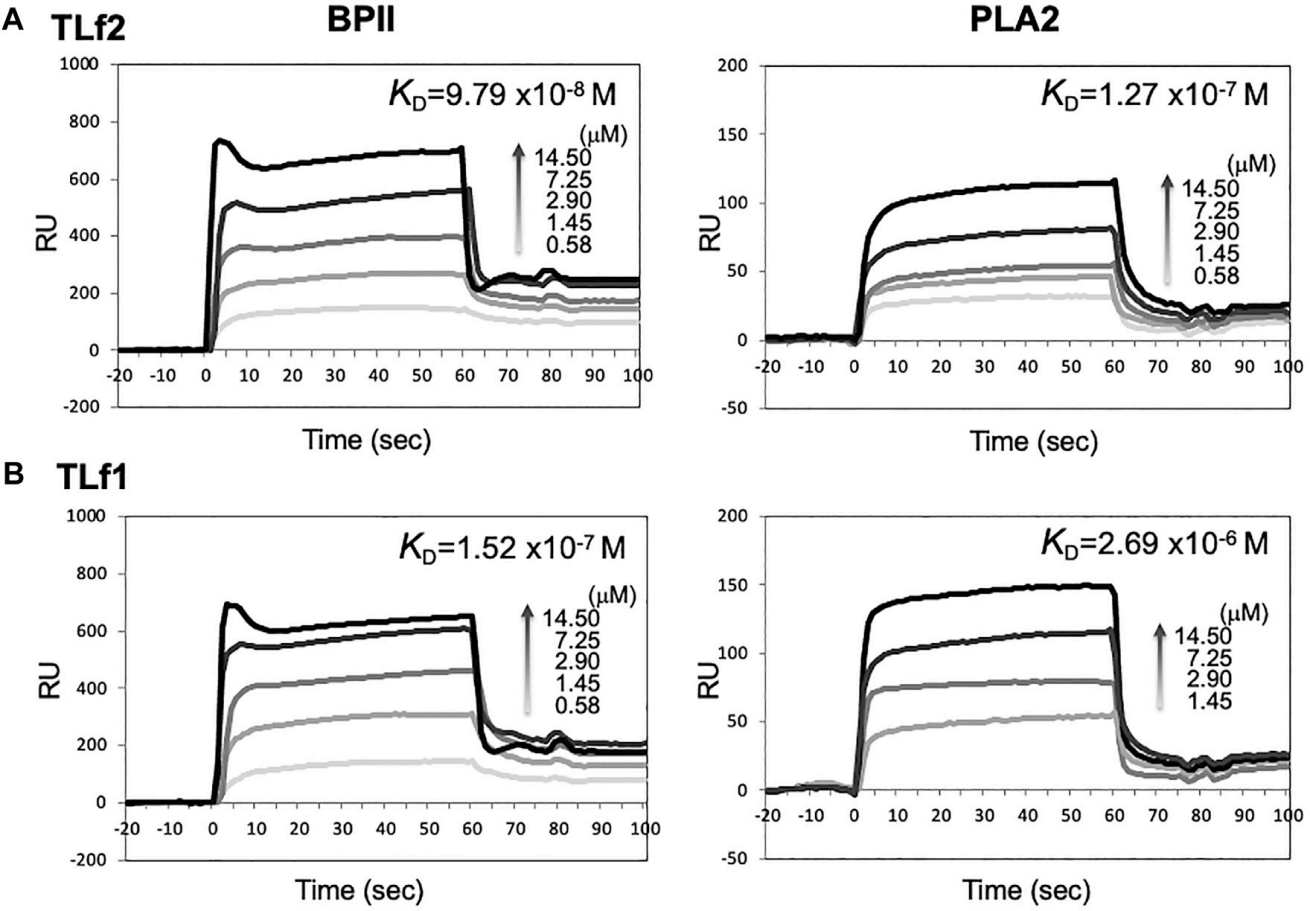
SPR sensorgrams of BPII **(left)** and [Asp49] PLA2 **(right)** binding to immobilized TLf2 **(A)** and TLf1 **(B)**, respectively. Surface plasmon resonance (SPR) were measured to determine the interaction between TLfs and PLA2s by using BIAcore X. Purified TLf2 and TLf1 were immobilized to CM5 sensor chip, respectively, and BPII and [Asp49] PLA2 were injected as analytes (12.5–100 μg/ml) at a flow rate of 20 μL/min. Sensorgrams were analyzed using nonlinear least squares curve fitting with BIAevaluation software.

**FIGURE 7:**
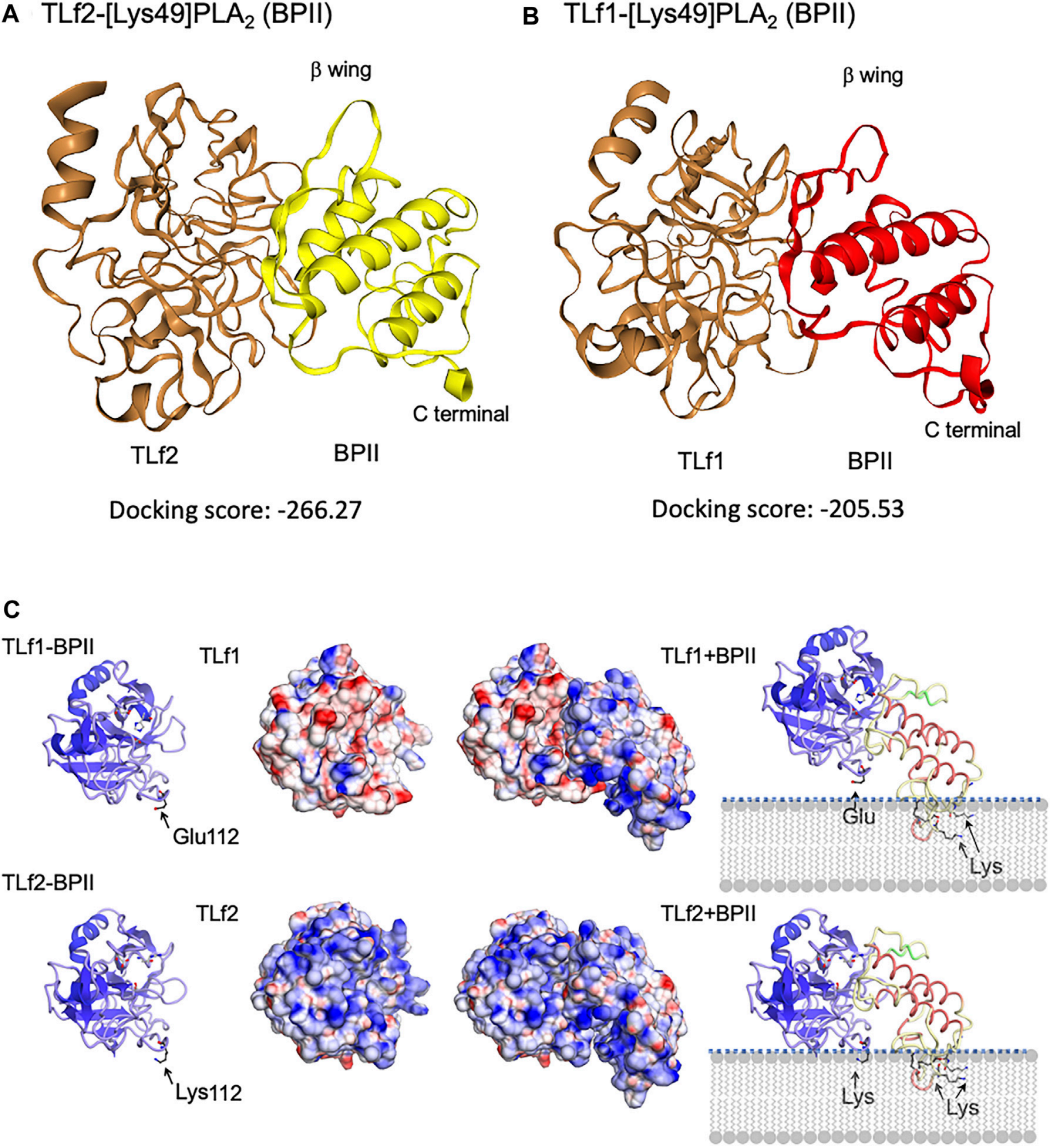
Predicted 3D structures of complex of BPII and TLf2 **(A)** or TLf1 **(B)**, and the surface electric charge of complexes, TLf1-BPII and TLf2-BPII **(C)**. Complex structures were estimated by the protein-protein docking simulation using HDOCK server that based on hybrid docking strategies, template-based modeling and *ab initio* docking (Yan et al., 2020). 3D structures of TLf1 and TLf2 were predicted by homology modeling based on the complex 3D structure of trypsin and its inhibitor (PDB 1TX6), while [Lys49] PLA2 (BPII, PDB 6AL3) was used as a ligand molecule. Predicted models with the lower docking energy score, entry no.1 for TLf2 and entry no. 3 for TLf1 (Supplementary Figure S6), were shown.

To elucidate the mechanisms underlying the synergistic effect of TLf2 on myonecrotic [Lys49] PLA2, direct interactions between TLfs and PLA2s were assessed via SPR. Figure 6 illustrates typical sensor grams of SPR for TLf2 and TLf1 with BPII or [Asp49] PLA2. TLf2 interacted with BPII at a nanomolar level (*K*_d_ = 9.79 × 10^−8^ M), while TLf1 desplayed weaker binding affinity (*K*_d_ = 1.52 × 10^−7^ M). Further, TLf2 and TLf1 displayed much weaker binding affinities for [Asp49] PLA2 than for BPII (Figure 6, Supplementary Table S3). These results indicated that the binding properties of the TLfs for BPII were correlated with their myonecrosis-enhancing activities. Furthermore, interactions between TLfs and BPII were assessed by simulating protein-protein docking *in silico* using HDOCK server. Figure 7 shows the typical predicted interaction mode between BPII and TLf2 (Figure 7A) or TLf1 (Figure 7B), demonstrating that both interactions predicted similar complex structures. However, TLf2 and TLf1 differed significantly with respect to their docking energy scores and their top 10 prediction models (Supplementary Figure S7). The docking energy scores for the top 10 models of the TLf2-BPII complex (average value 220.33) were more stable than those for the TLf1-BPII complex (average value 199.10). The top 10 models of the TLf2-BPII complex predicted the same sites of interaction between TLf2 and BPII (Supplementary Figure S7B), while those of the TLf1-BPII complex predicted two interaction sites that located on the opposite side (Supplementary Figure S7A). Further, structural model of complex of TLf2 and BPII indicated the obvious emphasized basic properties composed of positively charged amino acids, while TLf1-BPII showed the different electric charged properties compared with TLf2-BPII (Figure 7C). Thus, these *in silico* predictions explained the difference in binding affinities for TLf1 and TLf2, and the different properties such as surface charged distribution of complexes.

## Discussions

In this study, we compared two antivenoms, conventional antivenom (lot no. 34) and newly prepared antivenom (lot no. 1469). Histochemical analysis showed that the antivenom (lot no. 1469) prevented the necrosis of mouse fascia lata tissues, while lot no. 34 exerted no protective effects (Figure 1). The different properties between antivenom without anti-myonecrotic activity (lot no.34) and one with anti-myonecrotic activity (lot no.1469) are probably due to the different immunization strategies including numbers of booster and doses of venom (up to 320 mg), and also due to different antigens between Okinawa habu venom and Amami ones. Previously, we reported that the myotoxic [Lys^49^] PLA_2_ isoenzymes, BPI and BPII, which are expressed abundantly in the venoms of Amami-Oshima and Tokunoshima *P. flavoviridis,* are missing from the venom of Okinawa *P. flavoviridis* (Chijiwa et al., 2000; Chijiwa et al., 2003) due to the gene disruption of [Lys49] PLA2 encoding genes (Yamaguchi et al., 2015). These differences were supported by ELISA using two antivenoms, that is, the antivenom (lot no. 1469) with anti-myonecrotic activity showed the higher sensitivity against BPII compared with conventional antivenom (lot no. 34), although both antivenoms showed the similar reactivity against [Asp49] PLA2 (Supplementary Figure S6).

Furthermore, we applied a focused proteomics strategy to identify the catalytically inactive thrombin-like serine protease (TLSP2), TLf2, as a target protein of antiserum that prevents habu venom-induced necrosis caused by [Lys49] PLA2. As a result, it was revealed that a novel function of snake venom serine proteases–i.e., the synergistic myonecrosis-enhancing activity of TLf2 on [Lys49] PLA2, although TLf2 alone did not possess any cytotoxicity or no enzymatic activity as a serine protease. To our knowledge, this is the first report of a serine protease enhancing the toxicity of [Lys49] PLA2 myotoxins in snake venom.

To date, a large number of snake venom serine proteases have been sequenced and some have been functionally characterized (Ullah et al., 2018). These include pro-coagulant enzymes such as thrombin-like enzymes, including flavoxobin (TLf1) that clots fibrinogen, factor V, VIII, and XIII activators, kininogenases, plasminogen activators, platelet activators, and protein C activators (Seeger and Ouyang, 1979; Ullah et al., 2018). However, the functions of enzymatically inactive thrombin-like serine proteases remain unknown, although the cDNA and gene sequences of several inactive thrombin-like serine protease mutants have been determined, including TLf2 (Deshimaru et al., 1996; Wu et al., 2008; Vaiyapuri et al., 2011; Shibata et al., 2018). The serine protease TLf2 identified in the current study is an inactive proteolytic isoform due to the replacement of the active site, His43 with Arg.

More recently, genomic, transcriptomic, and bioinformatic analyses of many organisms have been confirmed that “pseudoenzymes”, which are catalytically inactive enzymes due to the loss of catalytic amino acid residue(s), are widely found in more than 20 enzyme superfamilies, and are classified into four groups based on their mode of functions: allosteric regulator, signal integrator/molecular switch, nucleate assembly of protein complexes, and competition for holoenzyme assembly (Adrain and Freeman, 2012; Murphy et al., 2017a; Murphy et al., 2017b; Ribeiro et al., 2019). In the case of snake venom, catalytically inactive subunits of neurotoxic PLA2 vipoxin are known to attenuate the toxicity of an active PLA2 paralog via heterodimerization (Banumathi et al., 2001). Mytotoxic [Lys49] PLA2s are also categorized as catalytically inactive PLA2s, of which the Ca^2+^ binding amino acid [Asp49] is replaced with Lys, and a synergism between [Asp49] and [Lys49] PLA2 myotoxins from *Bothrops asper* has been reported (Mora-Obando et al., 2014). These resarchers hypothesized that the toxicity-enhancing mechanism of [Lys49] PLA2 myotoxins exerted by [Asp49] PLA2 myotoxins may involve the weakening of cell membrane integrity by the latter, and the possible generation of new anionic sites. Synergistic action of a non-toxic PLA2 has been also reported in the enhancing the detachment of endothelial cells induced by a metalloproteinase (Bustillo et al., 2015). Although inactive thrombin-like serine proteases such as TLf2 can be categorized as pseudoenzymes, it was unknown whether they functioned as toxins on target tissues and cells as toxins until our study. Interestingly, TLf2 displayed much stronger binding affinity for BPII than TLf1, in accordance with the extent of the synergistic effects exerted by the two TLfs. These observations were further supported by the results of the protein-protein docking simulations. Lomonte and others have proposed that the myotoxic site of [Lys49] PLA2s is C-terminal basic region composed lysine cluster and permeabilized cell membrane via binding of C-terminal basic region to anionic site of phospholipids (Lomonte et al., 1994; Lomonte and Rangel, 2012). Structural model of complex of TLf2 and BPII indicated the obvious emphasized basic properties composed of positively charged amino acids including Lys112 of TLf2 (replaced by Glu in TLf1) and C-terminal toxic site (lysine cluster) of BPII, while TLf1-BPII showed the different electric charged properties compared with TLf2-BPII (Figure 7C). The synergistic mechanism was thus characterized in the TLf2 and [Lys49] PLA2 complex to be their positive charged properties potentiate the interaction via phospholipids membrane.

Furthermore, this study suggests that consideration should be given to the synergistic effects of toxins in the design and the development of antivenom and/or new treatments for snakebites.

## Data Availability

The datasets presented in this study can be found in online repositories. The names of the repository/repositories and accession number(s) can be found below: JPOST with accession JPST001309/PXD028215 and JPST001307.
