# Supplementary material for: Focused Proteomics Analysis of Habu Snake (Protobothrops flavoviridis) Venom Using Antivenom-Based Affinity Chromatography Reveals Novel Myonecrosis-Enhancing Activity of Thrombin-Like Serine Proteases
Source: Front Pharmacol. 2021 Nov 4;12:766406. doi: 10.3389/fphar.2021.766406 (PMC8599580; doi:10.3389/fphar.2021.766406)
Supplement: Supplementary file 1 [file DataSheet1.PDF]

Supplementary Material

1 Supplementary Figures and Tables

1.1 Supplementary Figures

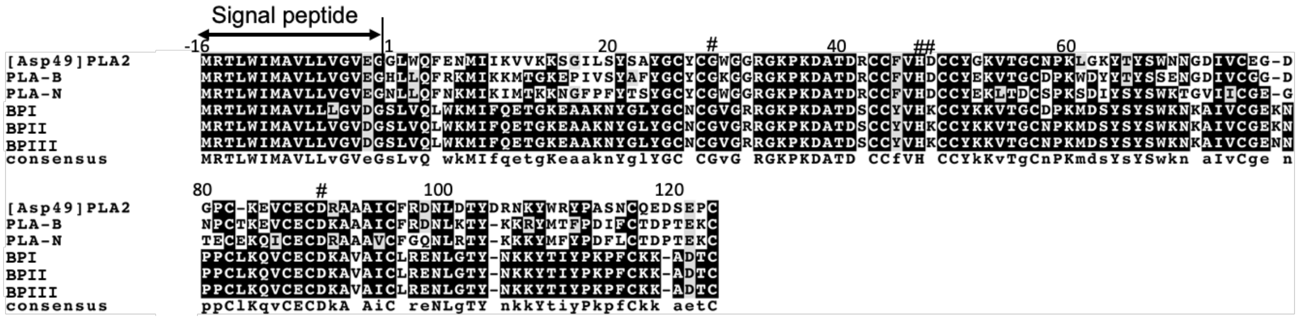

Supplementary Figure S1. Amino acid sequences of Protobothrops flavoviridis PLA2 isozymes. The sequences were aligned using the Clustal W program and represented by using BOXSHADE 3.21. The symbol # indicates the catalytic residues (His48 and Asp90), and calcium binding residues (Gly30 and Asp49).

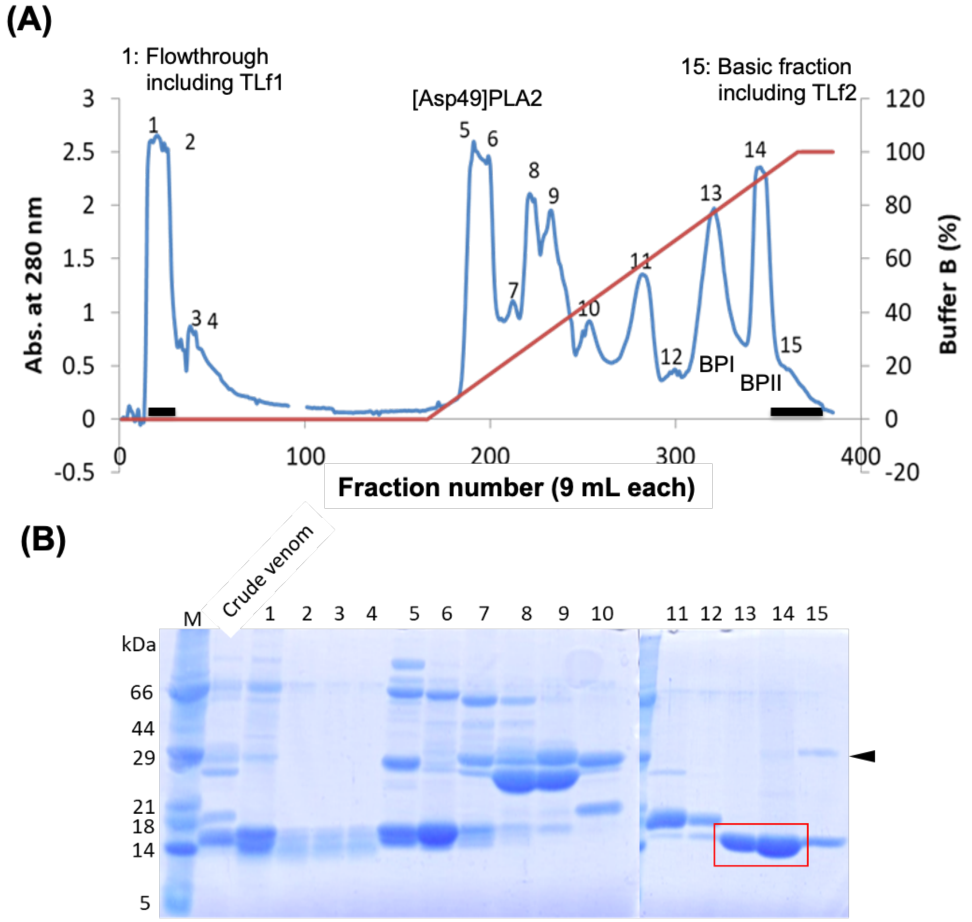

Supplementary Figure S2. Purification of *P. flavoviridis* crude venom on CM52 cation-exchange chromatography (A) and SDS-PAGE profiles of each peak fraction (B). The separation of crude venom (2.1 g) was achieved by cation-exchange chromatography on a CM52 column (ϕ15 mm × 870 mm) with a linear gradient from 20 mM CH_3COONH_4/0.1 mM $CaCl_2$ (pH6.8) (buffer A) to 500 mM CH_3COONH_4/0.1 mM $CaCl_2$ (pH6.8) (buffer B). Fractions 1 and 15 (indicated by black lines) contained TLf1 and TLf2, respectively, and were further purified as described in Fig. S3 and Fig. S4. SDS-PAGE was carried out on 15% gel. Lane numbers (1 to 15) correspond with the numbers in CM52 cation-exchange chromatography (A). The arrowhead indicated the 30 kDa protein, TLf2. Red squared bands indicate [Lys49]PLA2s, BPI and BPII.

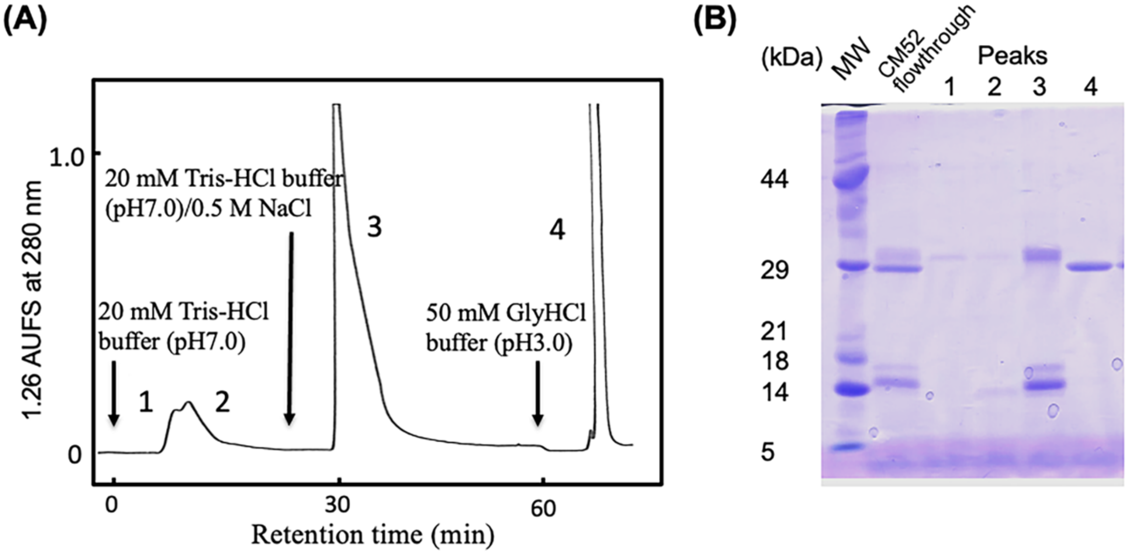

Supplementary Figure S3. Purification of TLf1 (flavoxobin) by affinity chromatography (A) and SDS-PAGE profiles of separated proteins (B). Flow-through fraction of *P. flavoviridis* crude venom on CM52 column (Fig. S1) was loaded on the Hitrap benzamidine column, washed with 20 mM Tris-HCl buffer (pH 7.0) containing 0.1 M NaCl, and subsequently eluted with 50 mM glycine HCl buffer.

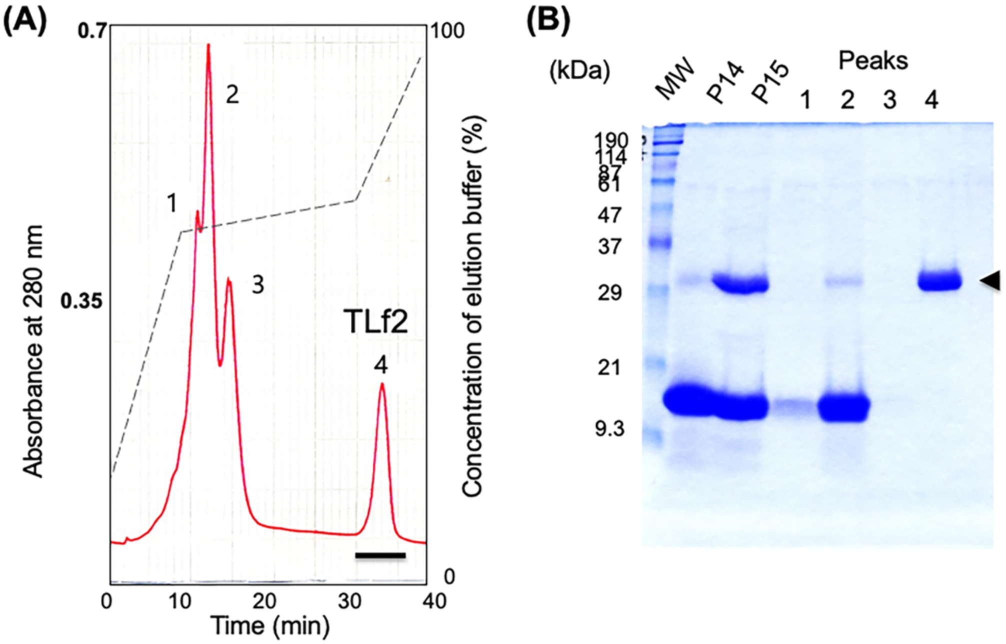

Supplementary Figure S4. Elution profile of basic fraction #15 using cation exchange chromatography (A) and SDS-PAGE profiles of purified TLf2 (B). Separation was achieved by the POROS HS column using 50 mM Tris-HCl (pH 8.8) buffer with a 0−1 M NaCl gradient. SDS-PAGE was carried out on 15% gel by loading fractions 1−4 from the POROS HS column, and fractions 14 (P14) and 15 (P15) from CM52 column chromatography (Fig. S1).

(A)

	%Cov	Names	Conf	Sequence	Modifications	Cleavages	dMass	Prec MW	Prec m/z	Theor MW	Theor m/z
1	12.6900002	flavoxobin; SVTLE	99.000001	FICPNKK	Carbamidomethyl(C)@3	missed K-K@6	-0.01771257	905.461609	906.4689	905.479309	906.486572
2	12.6900002	flavoxobin; SVTLE	99.000001	VLNEDEQIRNPK			-0.01942616	1453.73267	1454.74	1453.75256	1454.75977
3	12.6900002	flavoxobin; SVTLE	1.99999996	LDSPVSYSEHIAPL		cleaved L-S@C-term	0.01278799	1526.77466	1527.782	1526.76172	1527.76892
4	12.6900002	flavoxobin; SVTLE	99.000001	VLNEDEQIRNPK	Asn->Gln@3		0.001300727	1467.76965	1468.777	1467.76819	1468.77539
5	12.6900002	flavoxobin; SVTLE	80.0000012	VLNEDEQIRNPK	Asp->His@5		-0.06531102	1475.71863	1476.726	1475.78442	1476.79175

(B)

>prf||1405260A flavoxobin
VIGGDECNINEHPFLVALYDAWSGRFLCGGTLINPEWVLTAAHCDSKNFKMKLGAHSQK**VLNEDEQIRNPK**EK**FICPNKK**NTEVLDKDIMLIK
LDSPVSYSEHIAPLSLPSSPPSVGSVCRIMGWGSITPVEETFPDVPHCANINLLDDVECKPGYPELLPEYRTLCAGVLQGGIDTCGFDSGTPL
ICNGQFQGIVYIGSHPCGQSRKPGIYTKVFDYNAWIQSIIAGNTAATCLP

Supplementary Figure S5. Identification of TLf1 (flavoxobin) by MALDI-TOF-MS/MS analysis. MS/MS data for purified TLf1 (flavoxobin) (A) and the amino acid sequence of TLf1 (flavoxobin) (B). Bold-faced types indicate the peptide fragments determined by MS/MS analysis.

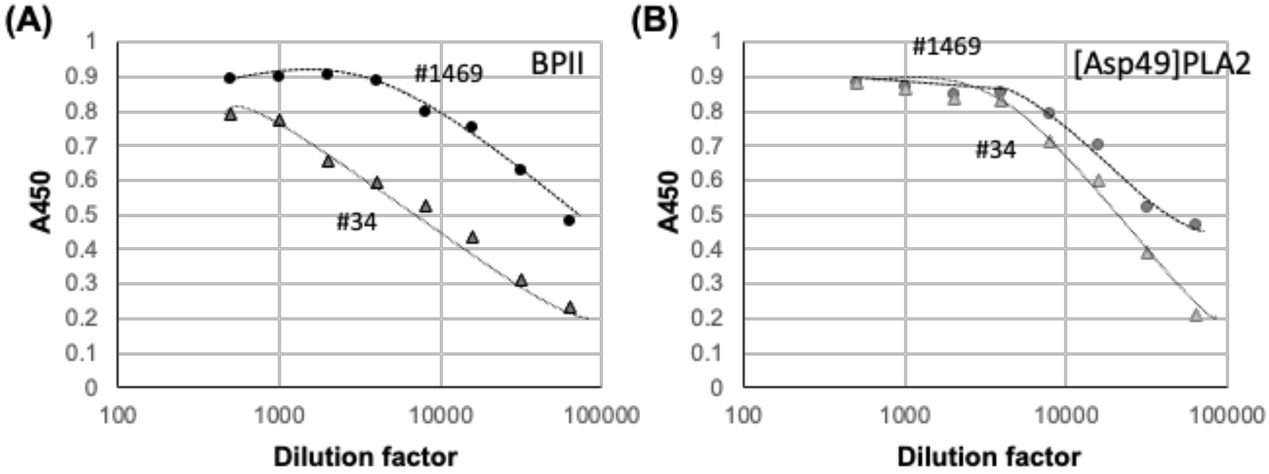

Supplementary Figure S6. Titration of two antivenoms (#34 and #1469) specificity to [Lys49]PLA2 (BPII) (A) and [Asp49]PLA2 (B) estimated by ELISA test.

(A)

N	%Cov	Names	Conf	Sequence	Modifications	Cleavages	dMass	Prec MW	Prec m/z	Theor MW	Theor m/z
1	72.4600017	BP-II; PLA2 i	99.000001	AVAICLREN	Carbamidomethyl(C)@5	cleaved N-L@C-term	0.0333332	1044.57166	1045.579	1044.53857	1045.5459
2	72.4600017	BP-II; PLA2 i	99.000001	AVAICLRENLGTYN	Carbamidomethyl(C)@5	cleaved N-K@C-term	0.06952532	1592.86768	1593.875	1592.7981	1593.8053
3	72.4600017	BP-II; PLA2 i	99.000001	AVAICLRENLGTYNK	Cys->Pro@5; Arg->Lys@7		0.02463472	1629.93372	1630.941	1629.90906	1630.91626
4	72.4600017	BP-II; PLA2 i	99.000001	AVAICLRENLGTYNKK	Carbamidomethyl(C)@5	missed K-K@15	0.09810513	1849.08569	1850.093	1848.98804	1849.99524
5	72.4600017	BP-II; PLA2 i	99.000001	DATDSCCYVHK	Carbamidomethyl(C)@6;		-0.0319217	1354.4967	1355.504	1354.5282	1355.53552
6	72.4600017	BP-II; PLA2 i	99.000001	GSLVQLWK		cleaved D-G@N-term	-0.0013	929.532166	930.5394	929.533447	930.54071
7	72.4600017	BP-II; PLA2 i	99.000001	ICLRENLGTYNK	Carbamidomethyl(C)@2	cleaved A-I@N-term	0.04908856	1479.79968	1480.807	1479.75037	1480.75769
8	72.4600017	BP-II; PLA2 i	99.000001	KYTIYPK		cleaved K-P@C-term; misse	0.00517829	911.516846	912.5241	911.511658	912.518921
9	72.4600017	BP-II; PLA2 i	99.000001	LRENLGTYNK		cleaved C-L@N-term	-0.0025811	1206.63269	1207.64	1206.63574	1207.64294
10	72.4600017	BP-II; PLA2 i	99.000001	MDSYSYSWK	Dethiomethyl(M)@1		0.01974095	1117.4917	1118.499	1117.47168	1118.47888
11	72.4600017	BP-II; PLA2 i	99.000001	MIFQETGK			0.02660603	952.495422	953.5027	952.468811	953.476074
12	72.4600017	BP-II; PLA2 i	99.000001	NYGLYGCNCGVGRRGK	Carbamidomethyl(C)@7;	cleaved K-P@C-term	0.03723304	1829.87866	1830.886	1829.84131	1830.84863
13	72.4600017	BP-II; PLA2 i	99.000001	NYGLYGCNCGVGRRGKPK	Carbamidomethyl(C)@7;		0.03830987	2055.02759	2056.035	2054.98901	2055.99634
14	72.4600017	BP-II; PLA2 i	99.000001	PKDATDSCCYVHK	Carbamyl(K)@2; Carbamidomethyl(C)@8; Carbamidomethyl(C)@9	cleaved K-P@N-term; misse	-0.0388557	1622.6427	1623.65	1622.68176	1623.68896
15	72.4600017	BP-II; PLA2 i	99.000001	YTIYPKPFCK	Carbamidomethyl(K)@6;		0.05790176	1372.74268	1373.75	1372.68494	1373.69226
16	72.4600017	BP-II; PLA2 i	98.0000019	SLVQLWK		cleaved G-S@N-term	0.0027077	872.514709	873.522	872.511963	873.519287
17	72.4600017	BP-II; PLA2 i	15.9999996	QVCECDK	Gln->pyro-Glu@N-term; Carbamidomethyl(C)@3;		-0.0292597	920.307556	921.3148	920.336792	921.344055
18	72.4600017	BP-II; PLA2 i	99.000001	AVAICLRENLGTYNK	Carbamidomethyl(C)@5		0.07380547	1720.96667	1721.974	1720.89307	1721.90027
19	72.4600017	BP-II; PLA2 i	99.000001	AVAICLRENLGTYNK	Carbamidomethyl(C)@5; Asn->Pro@14		0.1334271	1704.03662	1705.044	1703.90283	1704.91016
20	72.4600017	BP-II; PLA2 i	97.0000029	AVAICLRENLGTYNK	Carbamidomethyl(C)@5; Arg->Val@7		0.07427603	1663.93469	1664.942	1663.86035	1664.86768
21	72.4600017	BP-II; PLA2 i	9.00000036	AVAICLRENLGTYNK	Carbamidomethyl(C)@5; Leu->Met@6; Carbamyl(R)@7; Deamidated(N)@9		0.0485761	1782.8877	1783.895	1782.83923	1783.84656
22	72.4600017	BP-II; PLA2 i	0	AVAICLRENLGTYNK	Carbamidomethyl(C)@5; Dehydrated(T)@12		0.03273441	1702.91467	1703.922	1702.88245	1703.88977
23	72.4600017	BP-II; PLA2 i	99.000001	AVAICLRENLGTYNKK	Carbamidomethyl(C)@5; Carbamyl(K)@15; Lys->Arg@16	missed K-K@15	0.1337509	1920.13367	1921.141	1920	1921.0072
24	72.4600017	BP-II; PLA2 i	99.000001	AVAICLRENLGTYNKK	Carbamidomethyl(C)@5; Carbamidomethyl(K)@15	missed K-K@15	0.1075252	1906.1167	1907.124	1906.00952	1907.01672
25	72.4600017	BP-II; PLA2 i	99.000001	DATDSCCYVHK	Cys->Gln@6; Cys->Thr@7		-0.0749281	1263.49768	1264.505	1263.57312	1264.58044
26	72.4600017	BP-II; PLA2 i	98.0000019	DATDSCCYVHK	Asp->Gly@1; Thr->Asn@3; Carbamidomethyl(C)@6;		-0.0320673	1309.48572	1310.493	1309.51794	1310.52527
27	72.4600017	BP-II; PLA2 i	56.0000002	DATDSCCYVHK	Carbamidomethyl(C)@6; Carbamidomethyl(C)@7; His->Arg@10;		-0.1669706	1416.40967	1417.417	1416.57617	1417.5835
28	72.4600017	BP-II; PLA2 i	41.9999987	GSLVQLWK		cleaved D-G@N-term	4.89945698	934.432922	935.4402	929.533447	930.54071
29	72.4600017	BP-II; PLA2 i	5.99999987	KYTIYPK		cleaved K-P@C-term; misse	3.42119408	914.932861	915.9401	911.511658	912.518921
30	72.4600017	BP-II; PLA2 i	99.000001	MDSYSYSWK	Oxidation(M)@1		0.02414105	1181.49365	1182.501	1181.46997	1182.47717
31	72.4600017	BP-II; PLA2 i	98.0000019	MDSYSYSWK			0.0402959	1165.51563	1166.523	1165.47498	1166.4823
32	72.4600017	BP-II; PLA2 i	99.000001	MIFQETGK	Oxidation(M)@1		0.00489698	968.468628	969.4759	968.463745	969.471008
33	72.4600017	BP-II; PLA2 i	98.0000019	MIFQETGK	Dethiomethyl(M)@1		-0.0074377	904.458008	905.4653	904.465454	905.472717
34	72.4600017	BP-II; PLA2 i	9.00000036	NYGLYGCNCGVGRRGK	Carbamidomethyl(C)@7; Asn->Pro@8; Carbamidomethyl(C)@9	cleaved K-P@C-term	0.1277384	1812.97864	1813.986	1812.8512	1813.85852
35	72.4600017	BP-II; PLA2 i	2.99999993	NYGLYGCNCGVGRRGK	Tyr->Ala@5; Carbamidomethyl(C)@7; Deamidated(N)@8; Carbamidomethyl(C)@9	cleaved K-P@C-term	0.06917831	1738.86865	1739.876	1738.79919	1739.8064
36	72.4600017	BP-II; PLA2 i	99.000001	NYGLYGCNCGVGRRGKPK	Carbamidomethyl(C)@7; Carbamidomethyl(C)@9; Arg->Asp@14; Gly->Cys@15; Carbamidomethyl(C)@15		0.02956293	2116.95361	2117.961	2116.92407	2117.9314
37	72.4600017	BP-II; PLA2 i	95.9999979	NYGLYGCNCGVGRRGKPK	Carbamidomethyl(C)@7; Asn->Pro@8; Carbamidomethyl(C)@9		0.1590887	2038.15771	2039.165	2037.9989	2039.00623
38	72.4600017	BP-II; PLA2 i	95.9999979	SLVQLWK	Carbamyl@N-term	cleaved G-S@N-term	-0.0208672	915.496948	916.5042	915.517822	916.525085
39	72.4600017	BP-II; PLA2 i	87.9999995	SLVQLWK	Oxidation(W)@6	cleaved G-S@N-term	0.004192	888.511108	889.5184	888.506897	889.51416
40	72.4600017	BP-II; PLA2 i	5.99999987	SLVQLWK	Trp->Kynurenin(W)@6	cleaved G-S@N-term	0.00339855	876.510315	877.5176	876.506897	877.51416
41	33.5799992	BP-II; PLA2 i	99.000001	NYGLYGCNCGVGKRGK	Carbamidomethyl(C)@7; Cys->Pro@9	cleaved K-P@C-term; misse	0.01101151	1738.86865	1739.876	1738.8573	1739.86462
42	33.5799992	BP-II; PLA2 i	99.000001	AVAICLREN	Carbamidomethyl(C)@5	cleaved N-L@C-term	0.0333332	1044.57166	1045.579	1044.53857	1045.5459
43	33.5799992	BP-II; PLA2 i	99.000001	AVAICLRENLGTYN	Carbamidomethyl(C)@5	cleaved N-K@C-term	0.06952532	1592.86768	1593.875	1592.7981	1593.8053
44	33.5799992	BP-II; PLA2 i	99.000001	AVAICLRENLGTYNK	Carbamidomethyl(C)@5		0.07380547	1720.96667	1721.974	1720.89307	1721.90027
45	33.5799992	BP-II; PLA2 i	99.000001	AVAICLRENLGTYNK	Cys->Pro@5; Arg->Lys@7		0.02463472	1629.93372	1630.941	1629.90906	1630.91626
46	33.5799992	BP-II; PLA2 i	99.000001	AVAICLRENLGTYNK	Carbamidomethyl(C)@5; Asn->Pro@14		0.1334271	1704.03662	1705.044	1703.90283	1704.91016
47	33.5799992	BP-II; PLA2 i	97.0000029	AVAICLRENLGTYNK	Carbamidomethyl(C)@5; Arg->Val@7		0.07427603	1663.93469	1664.942	1663.86035	1664.86768
48	33.5799992	BP-II; PLA2 i	9.00000036	AVAICLRENLGTYNK	Carbamidomethyl(C)@5; Leu->Met@6; Carbamyl(R)@7; Deamidated(N)@9		0.0485761	1782.8877	1783.895	1782.83923	1783.84656
49	33.5799992	BP-II; PLA2 i	0	AVAICLRENLGTYNK	Carbamidomethyl(C)@5; Dehydrated(T)@12		0.03273441	1702.91467	1703.922	1702.88245	1703.88977
50	33.5799992	BP-II; PLA2 i	99.000001	ICLRENLGTYNK	Carbamidomethyl(C)@2	cleaved A-I@N-term	0.04908856	1479.79968	1480.807	1479.75037	1480.75769
51	33.5799992	BP-II; PLA2 i	99.000001	LRENLGTYNK		cleaved C-L@N-term	-0.0025811	1206.63269	1207.64	1206.63574	1207.64294
52	33.5799992	BP-II; PLA2 i	2.99999993	NYGLYGCNCGVGKRGK	Carbamidomethyl(C)@7; Carbamidomethyl(C)@9; Lys->Arg@13	cleaved K-P@C-term; misse	0.03723304	1829.87866	1830.886	1829.84131	1830.84863
53	33.5799992	BP-II; PLA2 i	0.99999998	NYGLYGCNCGVGKRGK	Carbamidomethyl(C)@7; Carbamidomethyl(C)@9; Gly->Phe@15	cleaved K-P@C-term; misse	-0.0752244	1891.80664	1892.814	1891.8822	1892.8894
54	33.5799992	BP-II; PLA2 i	15.9999996	QVCECDK	Gln->pyro-Glu@N-term; Carbamidomethyl(C)@3;		-0.0292597	920.307556	921.3148	920.336792	921.344055

(B)

BPII

1 _mrtlwimavl lvgvd_**gslvq lwkmifqetg k**eaak**nygly gcncgvgrrg kpkdatdscc**

61 **yvhk**ccykkv tgcnpk**mdsy syswk**nkaiv cgeknppclk **qvcecdkava iclrenlgty**

121 **nkkytiypkp fck**kadtc

Supplementary Figure S7. Identification of [Lys49]PLA2 (BPII) by MALDI-TOF-MS/MS analysis. (A) MS/MS data for spot no. 2 on 2D PAGE (Fig. 2A) correspond to BPII. (B) Amino acid sequence of BPII. Bold-faced types indicate the sequences of peptide fragments (underlined) determined by MS/MS analysis.

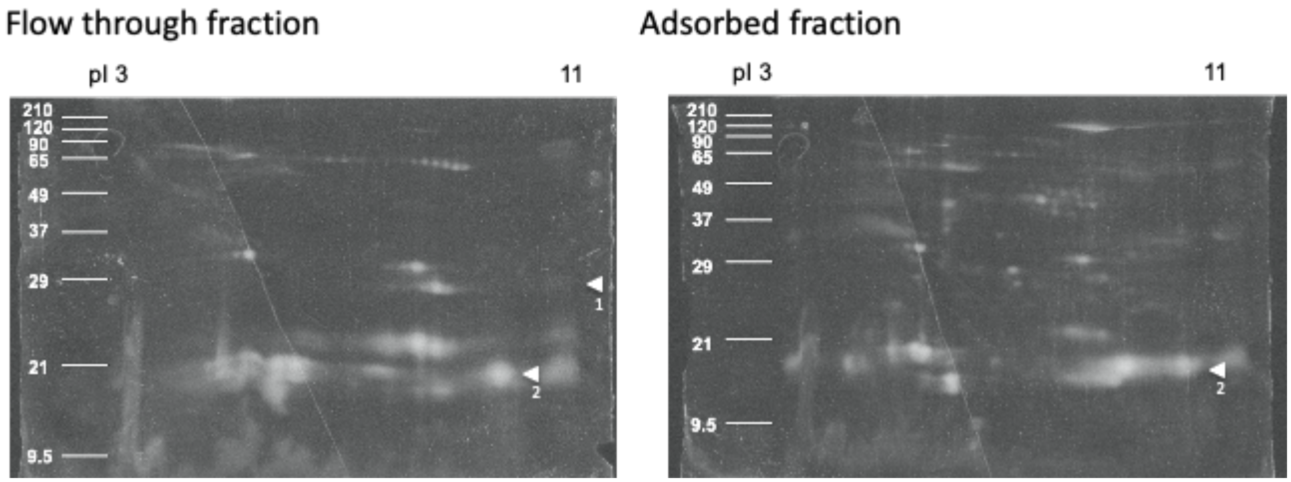

Supplementary Figure S8. 2D PAGE of habu crude venom separated by antivenom (AV)-conjugated affinity chromatography. 2D PAGE profiles of flow through (left) and adsorbed fractions (right) on antivenom-conjugated column using lot no. 34 antivenom. Samples were loaded on the IEF using the IPGphor System with immobilized pH gradient (IPG) strips (pI 3−11), and then subjected to the sodium dodecyl sulfate polyacrylamide gel electrophoresis (SDS-PAGE) under reducing conditions. SDS-PAGE was performed on 15% slab gels according to the method described by Laemmli. The gels were stained with Oriole fluorescent gel staining. Arrow heads 1 and 2 indicate TLf2 and BPII, respectively.

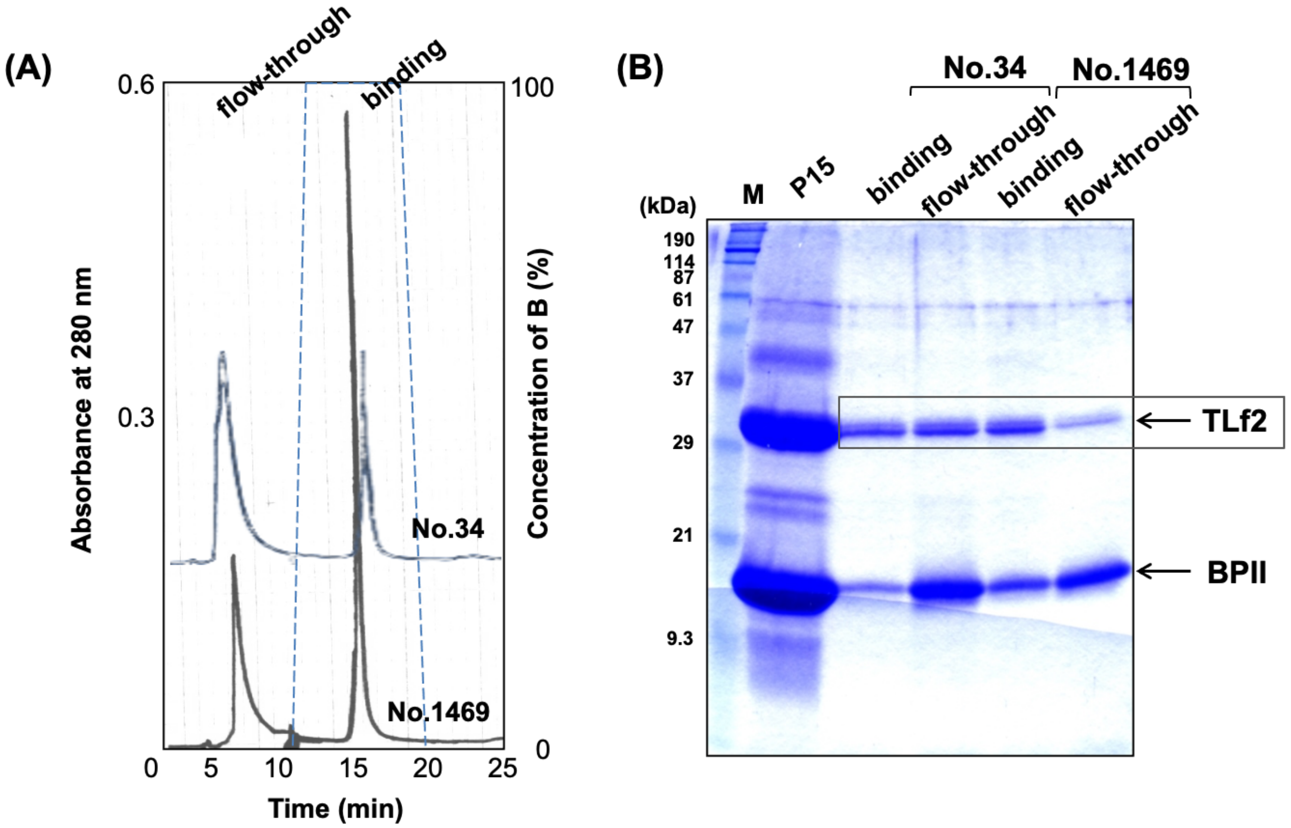

Supplementary Figure S9. Comparison of affinity chromatograms (A) and SDS-PAGE profiles (B) of basic fraction containing TLf2 and BPII between antivenom-specific-conjugated columns (no. 34 without anti-myonecrotic activity; no. 1469 with anti-necrotic activity). Basic fraction #15 (P15) on CM52 cation-exchange chromatography containing TLf2 was used for affinity chromatography. The proteins bound to antivenom-specific-conjugated affinity columns were eluted with 50 mM glycine HCl buffer (pH 3.0). SDS-PAGE was carried out on 15% gel.

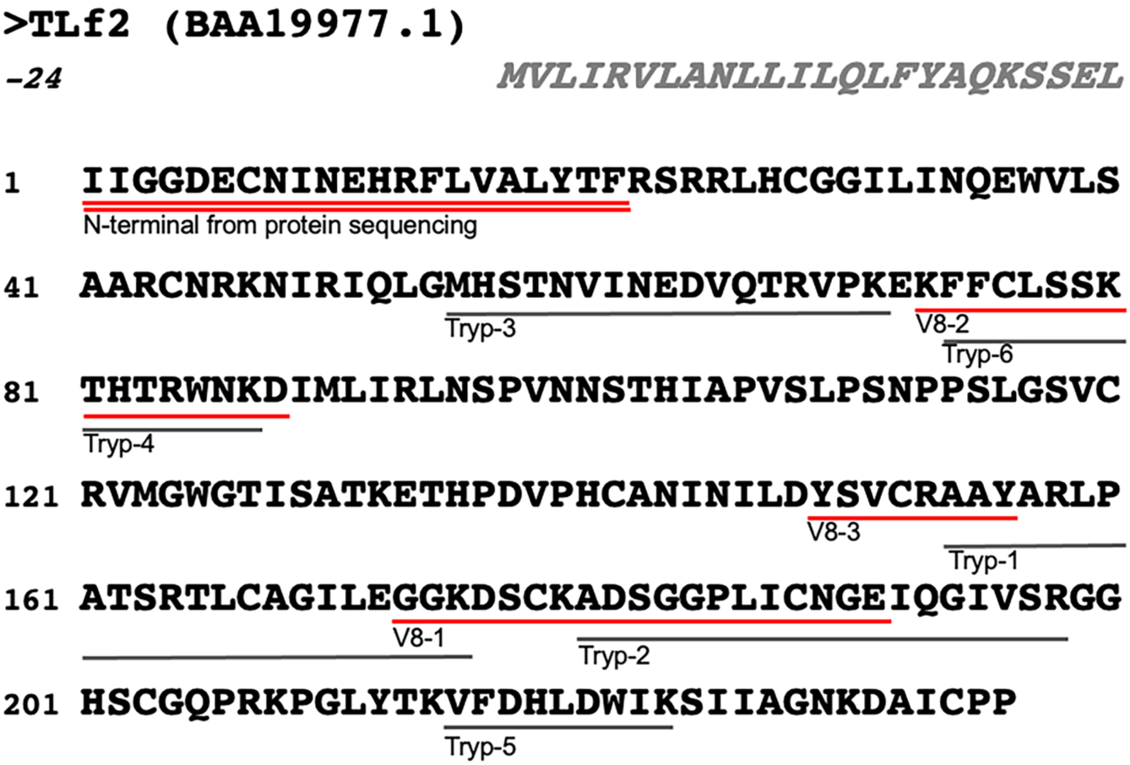

Supplementary Figure S10. Identification of basic 30 kDa protein as TLf2 by amino acid sequence analysis using combined MALDI-TOF MS/MS and N-terminal protein sequencing. The double red line indicates the N-terminal amino acid sequence determined by gas-phase Edman degradation for protein sequencing. Single lines indicate the amino acid sequences identified by MALDI-TOF MS/MS analysis with V8 protease (V8-1 to V8-3) and trypsin (Tryp-1 to Tryp-6) digestions, respectively. Sequence data is available with the accession number of BAA19977.1 (D67079.1).

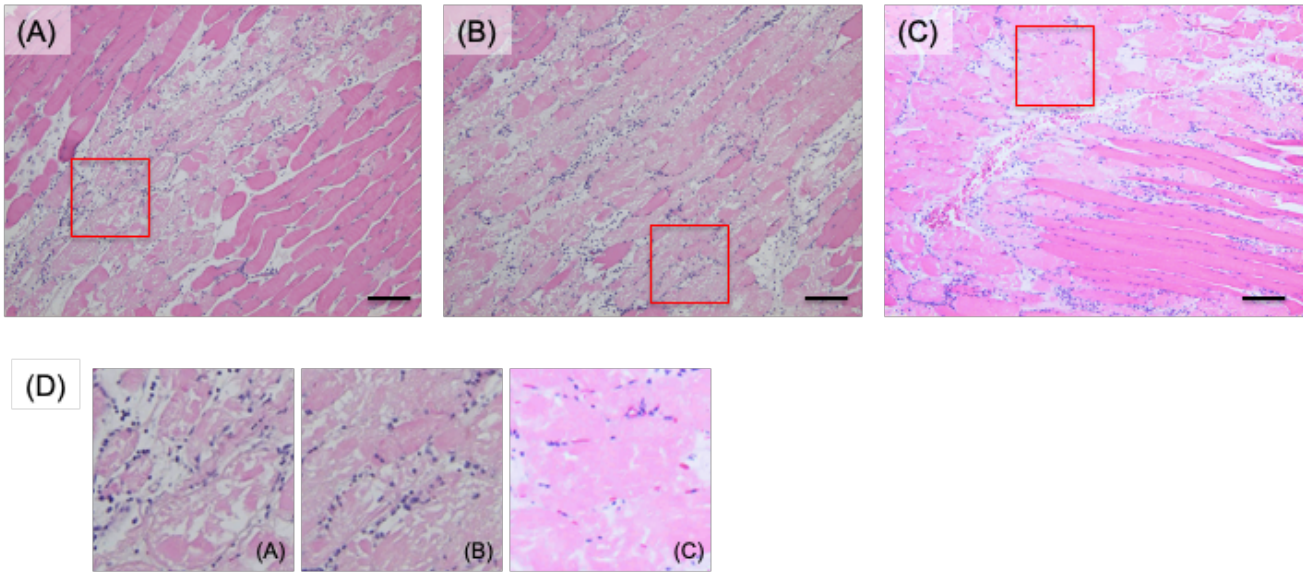

Supplementary Figure S11. Synergistic cytotoxic effects of TLf2 (A) and TLf1(B) against BPII compared with BPII alone (C). Typical necrosis lesions of muscle tissue (red squared) were magnified and compared (D). Damages in necrotic muscle tissues were estimated by the area of voidage. Images (A), (B), and (C) are the same magnification. Scale bar = 100 µm.

(A) TLf1-Lys49PLA2 (BPII)

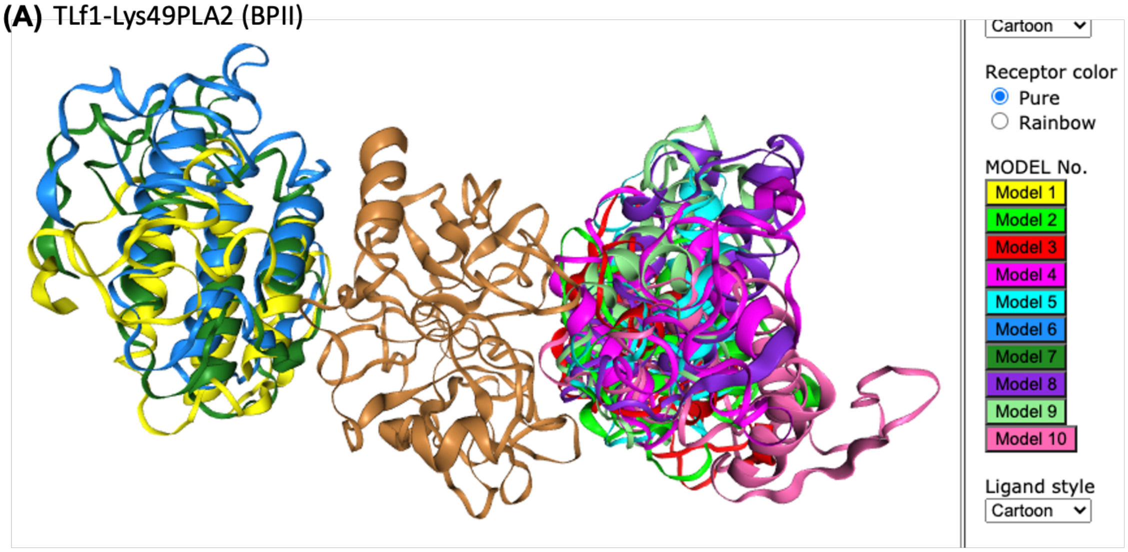

Summary of the Top 10 Models

Rank	1	2	3	4	5	6	7	8	9	10
Docking Score	-205.80	-205.61	-205.53	-203.06	-200.43	-197.65	-194.76	-193.92	-192.95	-191.31
Ligand rmsd (Å)	62.76	65.42	65.09	47.93	58.14	60.37	62.73	52.90	57.38	53.54
Interface residues	model_1	model_2	model_3	model_4	model_5	model_6	model_7	model_8	model_9	model_10

(B) TLf2-Lys49PLA2 (BPII)

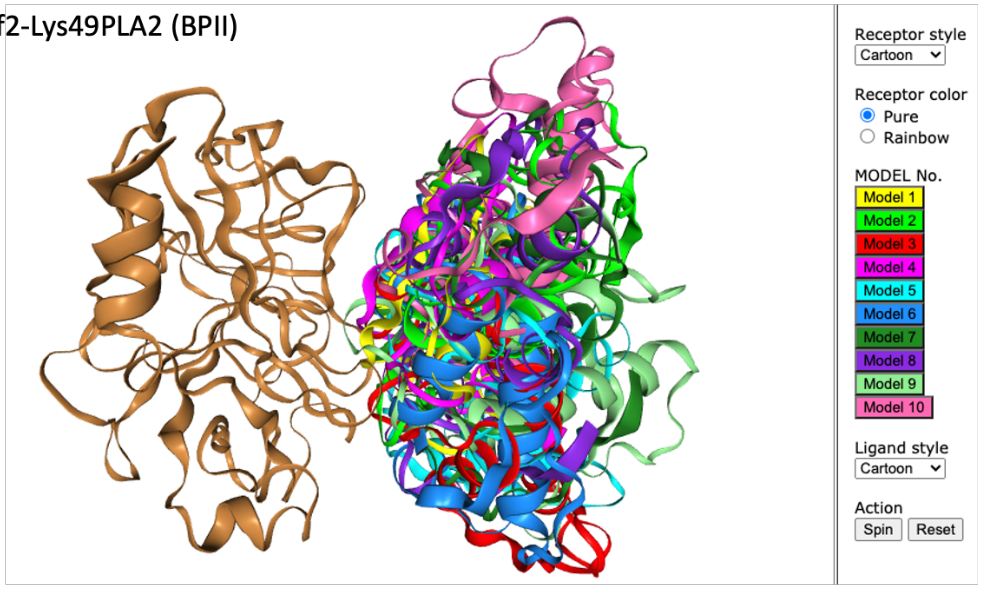

Summary of the Top 10 Models

Rank	1	2	3	4	5	6	7	8	9	10
Docking Score	-266.27	-236.55	-231.58	-217.86	-216.94	-216.68	-208.88	-207.23	-201.18	-200.09
Ligand rmsd (Å)	62.76	59.86	51.39	60.95	54.10	53.51	54.60	53.85	53.00	60.86
Interface residues	model_1	model_2	model_3	model_4	model_5	model_6	model_7	model_8	model_9	model_10

Supplementary Figure S12. Docking simulation of myonecrotic [Lys49] PLA2 (BPII) and serine proteases, TLf1 (A) or TLf2 (B). The top 10 models of the complex interaction between BPII and the serine proteases, TLf1 and TLf2, were estimated by protein-protein docking simulation using the HDOCK server that based on hybrid docking strategies, template-based modeling and *ab initio* docking.

1.2 Supplementary Tables

Supplementary Table S1 *P. flavoviridis* PLA2 isozymes

	Type	Activity	GenBank ID
[Asp49]PLA2	Asp49	hemolytic	D10070.1/D10720.1
PLA-B	Asp49	edema-inducing	D10721.1
PLA-N	Asp49	weak neurotoxin	AB848131
BPI	Lys49	myonecrosis	D10718.1
BPII	Lys49	myonecrosis	D10719.1
BPIII	Lys49	myonecrosis	AB470470.1

Supplementary Table S2 MALDI-TOF MS/MS analysis of 30 kDa protein, TLf2.

No.	Sequences from MS/MS	Precursor Mass	Theoretical Mass	ΔMass	Confidence
Tryp-1	AAYARLPATSRTLCAGILEGGK	2275.1826	2275.2107	-0.0283	99.00
Tryp-2	ADSGGPLICNGEIQGIVSR	1941.9266	1941.9578	-0.0309	99.00
Tryp-3	MHSTNVINEDVQTRVPK*	1966.9647	1966.9895	-0.0244	99.00
Tryp-4	THTRWNK	941.4544	941.4832	-0.0288	99.00
Tryp-5	VFDHLDWIK	1171.5547	1171.6025	-0.0482	99.00
Tryp-6	FFCLSSK	887.4163	887.4211	-0.0049	96.00
V8-1	GGKDSCKADSGGPLICNGE	1921.8877	1921.8146	0.0731	99.00
V8-2	KFFCLSSKTHTRWNKD	2086.2527	2086.0054	0.2473	99.00
V8-3	YSVCRAAY*	988.5371	988.4437	0.0934	59.00

Tryp-1~6: trypsin digested peptides, V8-1~3: V8 protease digested peptides.

* Undesired cleavage was occurred.

Supplementary Table S3 Kinetic parameters for interaction between TLfs and PLA2.

		ka(1/Ms)	kd(1/s)	R_{max} (RU)	K_A (1/M)	K_D (M)	Chi2
TLf1	BPII	1.69×10^{4}	2.57×10^{-3}	213	6.56×10^{6}	1.52×10^{-7}	213
	PLA2	4.66×10^{3}	0.0125	38.9	3.71×10^{5}	2.69×10^{-6}	3.43
TLf2	BPII	1.25×10^{4}	1.22×10^{-3}	242	1.02×10^{7}	9.79×10^{-8}	379
	PLA2	6.25×10^{3}	7.91×10^{-4}	14.2	7.90×10^{6}	1.27×10^{-7}	1.67
